# Tripartite Motif Containing 65 Deficiency Confers Protection Against Acute Kidney Injury via Alleviating Voltage‐Dependent Anion Channel 1–Mediated Mitochondrial Dysfunction

**DOI:** 10.1002/mco2.70149

**Published:** 2025-04-22

**Authors:** Tao Chen, Yang Zhang, Liting Ding, Chenlu Xiong, Chao Mei, Sisi Wei, Ming Jiang, Yingjie Huang, Jianrong Chen, Tao Xie, Qing Zhu, Qi Zhang, Xuan Huang, Shibiao Chen, Yong Li

**Affiliations:** ^1^ Department of Anesthesiology, The First Affiliated Hospital, Jiangxi Medical College Nanchang University Nanchang China; ^2^ The National Engineering Research Center for Bioengineering Drugs and the Technologies, Jiangxi Provincial Key Laboratory of Bioengineering Drugs, Institute of Translational Medicine, Jiangxi Medical College Nanchang University Nanchang China; ^3^ Department of Anesthesiology, Sir Run Run Shaw Hospital, School of Medicine Zhejiang University Hangzhou China; ^4^ Department of Endocrinology, The First Affiliated Hospital, Jiangxi Medical College Nanchang University Nanchang China

**Keywords:** acute kidney injury (AKI), mitochondrial dysfunction, tripartite motif containing 65 (TRIM65), ubiquitination, voltage‐dependent anion channel 1 (VDAC1)

## Abstract

Acute kidney injury (AKI) is a prevalent and serious clinical disease with a high incidence rate and significant health burden. The limited understanding of the complex pathological mechanisms has hindered the development of efficacious therapeutics. Tripartite motif containing 65 (TRIM65) has recently been identified as a key regulator of acute inflammation. However, its role in AKI remains unclear. The present study observed that TRIM65 expression was upregulated in AKI. Moreover, the knockout of the *Trim65* gene in mice exhibited a substantial protective impact against rhabdomyolysis, ischemia‐reperfusion (I/R), and cisplatin‐induced AKI. Mechanistically, TRIM65 directly binds and mediates K48/K63‐linked polyubiquitination modifications of voltage‐dependent anion channel 1 (VDAC1) at its K161 and K200 amino acid sites. TRIM65 plays a role in maintaining the stability of VDAC1 and preventing its degradation by the autophagy pathway. TRIM65 deficiency attenuates mitochondrial dysfunction in renal tubular epithelial cells during AKI. Conversely, the overexpression of VDAC1 in renal tissues has been demonstrated to negate the protective effect of TRIM65 deficiency on AKI. These findings suggest that TRIM65 may play a role regulating of AKI through the targeting of VDAC1‐dependent mitochondrial function, offering potential avenues for the development of new drug targets and strategies for the treatment of AKI.

## Graphical abstract

In the context of acute kidney injury (AKI) resulting from a multiplicity of causes, including chemotherapeutic agents such as cisplatin, I/R injury, and rhabdomyosarcoma, the TRIM65 protein is upregulated in renal tubular epithelial cells. The TRIM65 protein mediates K48/K63‐linked ubiquitination of the mitochondrial outer membrane protein VDAC1, thereby increasing its stability and resulting in mitochondrial dysfunction. In the absence of TRIM65, VDAC1 is maintained at a lower level of protein through the autophagy pathway, which promotes the repair of mitochondrial function and ultimately protects against acute kidney injury.

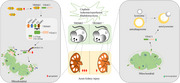

## 1 Introduction

Acute kidney injury (AKI) is a prevalent clinical syndrome typified by a precipitous decline in glomerular filtration rate (GFR), a swift elevation in serum creatinine (sCr) and blood urea nitrogen (BUN) levels, and a reduction in urine output [1]. The global incidence of AKI is estimated to be between 10% and 15% in hospitalized patients and often exceeds 50% in intensive care units (ICUs) [2]. It is estimated that AKI affects over 13 million individuals worldwide, resulting in 1.7 million deaths annually. Even in cases of mild AKI, the risk of mortality is 50% higher [3]. The global burden of AKI‐related mortality is significantly higher than that of other major diseases, including breast cancer, heart failure, and diabetes. Furthermore, the mortality rate has remained consistently high over the past five decades [4]. Despite significant advances in our understanding of AKI, there remains a dearth of efficacious therapeutic strategies for clinical application. It is therefore of the utmost importance to investigate the pathogenesis of AKI to identify effective treatment options.

It is established that certain pathways play a role in regulating AKI. For instance, PAFAH2 has been shown to suppress ferroptosis, thereby ameliorating AKI [5]. WWP2 deletion has been observed to target the CDC20/autophagy axis, leading to exacerbated AKI [6]. In recent years, E3 ubiquitin ligase has emerged as a potential therapeutic target due to its selective ability to connect ubiquitin to substrate proteins within the ubiquitination cascade, while also regulating cellular homeostasis [7]. The tripartite motif containing (TRIM) protein family is a group of RING‐type E3 ubiquitin ligases that have been linked to a multitude of cellular processes, including intracellular signaling, inflammation, and metabolism [8, 9]. The TRIM protein family comprises more than 80 members present in humans, which exhibit a conserved structural arrangement of three N‐terminal domains: a RING domain, one or two B‐box motifs, and the α‐helical coiled‐coil domain followed by a highly variable carboxyl‐terminal domain [8]. The precise function and mechanisms of TRIM proteins in AKI remain largely unelucidated.

The present study identified the upregulation of several TRIM proteins in both glycerol‐ and ischemia‐reperfusion (I/R)‐induced AKI mouse models. Of the proteins examined, TRIM65 exhibited the most significant differential changes. Recent evidence suggests that TRIM65 plays a significant role in the pathogenesis of acute inflammation. TRIM65 has been demonstrated to inhibit endothelial cell inflammation by binding to and ubiquitinating VCAM‐1, thereby promoting its degradation [10, 11]. Additionally, TRIM65 has been shown to target NLPR3 polyubiquitination, which may contribute to the inhibition of neuroinflammation [12, 13]. Additionally, we previously reported that depletion of TRIM65 can exacerbate intestinal I/R injury, primarily through promotion of TOX4‐dependent apoptosis [14]. Most recently, we also demonstrated that TRIM65 deficiency alleviates renal fibrosis through NUDT21‐mediated alternative polyadenylation [15]. However, its role in other acute inflammations, such as AKI, has not yet been reported.

The utilization of *Trim65* gene knockout (*Trim65* ^−/−^) mice to establish three distinct AKI mouse models revealed that TRIM65 deficiency significantly ameliorates AKI. The yeast two‐hybrid system was employed to identify voltage‐dependent anion channel 1 (VDAC1) as a potential novel target protein for TRIM65. TRIM65 has been demonstrated to bind directly to and mediate both the K48‐ and K63‐linked ubiquitination of VDAC1, thereby enhancing its protein stability. In AKI, the knockout of TRIM65 results in the downregulation of VDAC1, which subsequently minimizes mitochondrial dysfunction. This study, from a novel perspective, revealed that the depletion of TRIM65 protects against AKI by preserving mitochondrial function. This provides a potential new strategy and drug target for the prevention and treatment of AKI.

## 2 Results

### 2.1 The Expression of TRIM65 Is Increased in AKI

**FIGURE 1:**
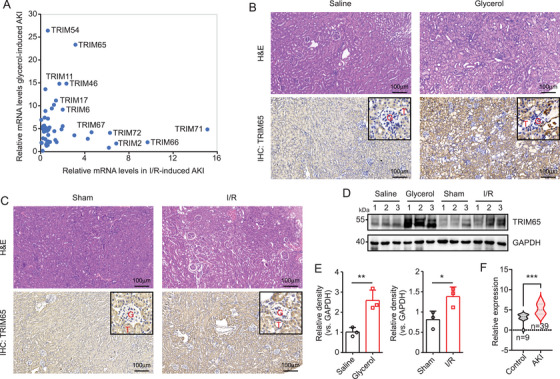
TRIM65 expression levels in rhabdomyolysis‐ and I/R‐ induced AKI. (A) WT C57BL/6 mice were utilized to establish rhabdomyolysis‐ and I/R‐induced AKI. The relative mRNA levels of the entire TRIM family in kidney tissues were quantified using RT‐qPCR with β‐actin serving as the internal reference. (B) Representative images of H&E and IHC staining of WT mice kidneys in saline and glycerol groups are presented, with the following scale bars: 100 µm. (C) Representative images of H&E and IHC staining of WT mice kidneys in the sham and I/R groups are presented. Scale bars: 100 µm. (D) The expression of TRIM65 in the kidneys of WT mice from glycerol‐ and ischemia/reperfusion‐induced AKI was detected by western blot, with GAPDH as the loading control. (E) The TRIM65 western blot band was quantified using ImageJ software. (F) The expression matrix was downloaded from the GEO dataset (GSE139061), which encompasses data from nine reference nephrectomies and 39 human AKI samples. Following normalization using the edgeR package, the differential expression of TRIM65 was analyzed using the Wilcoxon test. Statistical significance was indicated by *p < 0.05, **p < 0.01, ***p < 0.001.

To investigate the role of TRIM proteins in AKI, two mouse models were established: an I/R and a rhabdomyolysis‐induced AKI model. The expression levels of TRIM family members in renal tissues from both models were quantified by RT‐qPCR. As illustrated in Figure 1A, numerous TRIM family members exhibited a striking upregulation, with TRIM65 expression being particularly pronounced. In the renal tissue of rhabdomyolysis‐induced AKI, TRIM65 expression increased 23‐fold, while in I/R‐induced AKI renal tissue, it increased threefold. Histological examination by hematoxylin and eosin (H&E) staining revealed the development and progression of proximal tubular dilation, epithelial simplification, inflammatory cell infiltration, and cast formation in the renal tissue of the glycerol injection group in comparison to the saline group (Figure 1B). Immunohistochemical (IHC) staining using TRIM65‐specific antibodies indicated a significant upregulation of TRIM65 in renal tissue of AKI mice induced by rhabdomyolysis, with a predominant distribution in renal tubular epithelial cells, and no significant difference observed in glomeruli (Figure 1B). Similarly, extensive injury to the renal tubular epithelium was observed in I/R‐induced AKI mice, which was characterized by tubular dilation and swelling, cast formation, and interstitial inflammatory cell infiltration (Figure 1C). Moreover, IHC staining showed a significant increase in TRIM65 expression in tubular cells following I/R surgery when compared to the sham group (Figure 1C). Western blot analysis indicated a notable elevation in TRIM65 expression in the kidneys of mice subjected to rhabdomyolysis and I/R‐induced AKI models (Figure 1D,E). Furthermore, to investigate whether TRIM65 expression is also elevated in human AKI samples, the expression matrix was downloaded from the GEO dataset (GSE139061), which includes data from nine reference nephrectomies and 39 human AKI samples. Following normalization using the edgeR package, the expression of TRIM65 was found to be significantly increased in AKI samples, as determined by the Wilcoxon test (*p* < 0.001, Figure 1F). These findings indicate that TRIM65 is induced in AKI.

### 2.2 Genetic Deletion of TRIM65 Protected AKI in Experimental Mice

**FIGURE 2:**
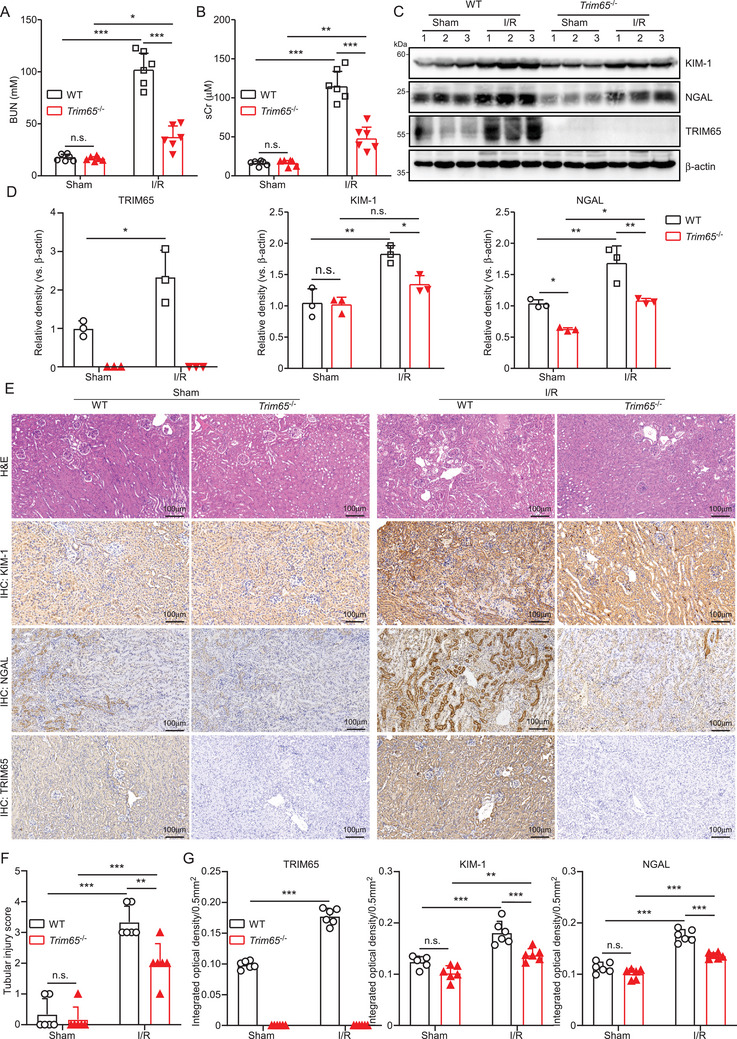
TRIM65 deficiency protected against I/R‐induced AKI. (A) The impact of TRIM65 deficiency on serum BUN levels in mice subjected to renal I/R or sham surgery for 24 h. (B) The impact of TRIM65 deficiency on sCr levels in mice undergoing renal I/R or sham surgery. (C) Following 40 min of ischemia and a subsequent 24‐h reperfusion period, kidney tissue homogenates were collected from the mice. Western blot analysis was conducted on the tissue proteins. (D) The western blot bands for KIM‐1, TRIM65, and NGAL were quantified. (E) Representative H&E staining of kidney sections and IHC staining detection of TRIM65, KIM‐1, and NGAL in four groups of mice. Following renal I/R or sham surgery in four groups of mice, one side of the kidney tissue was obtained and fixed with 4% paraformaldehyde 24 h after reperfusion. Scale bar: 100 µm. (F) The renal tubular injury score. (G) The degree of staining and the positive range of TRIM65, KIM‐1, and NGAL are evaluated under an optical microscope, and the IHC is scored accordingly. The number of mice per group was six. All data were subjected to a two‐way ANOVA and Tukey's post hoc test were employed for multiple group comparisons. Statistical significance was indicated by *p < 0.05, **p < 0.01, ***p < 0.001; n.s. indicates no significance.

To ascertain the function of TRIM65 in AKI, we initially assessed the impact of TRIM65 gene deletion on renal function and pathological alterations in rhabdomyolysis, I/R, and cisplatin‐induced mouse models of AKI. Serum levels of urea nitrogen and creatinine (BUN and sCr) were markedly elevated in WT mice with AKI, whereas TRIM65 deletion resulted in a notable decline in these levels (*p* < 0.001, Figure 2A,B, see also Figures S1A,B and S2A,B). Western blot analysis showed that two of the most utilized markers of AKI, kidney injury molecule 1 (KIM‐1) and neutrophil gelatinase‐associated lipocalin (NGAL), were found to be significantly upregulated in the renal tissue of WT mice that bad been induced to develop AKI. In contrast, these markers were significantly inhibited in the renal tissues of *Trim65* ^−/−^ mice (Figure 2C,D, see also Figures S1C,D, S2C, S3A). Histological examination of mouse renal tissues revealed that TRIM65 deficiency resulted in the attenuation of AKI (Figure 2E,F, see also Figures S1E,F and S2D). IHC staining suggests that the expression of KIM‐1 and NGAL was elevated in AKI mice. Consequently, the elevated protein level of KIM‐1 and NGAL observed after I/R was markedly decreased by TRIM65 deletion (Figure 2E,G, see also Figures S1E,G and S2D). Additionally, the mRNA expression levels of the inflammatory factors MCP‐1, IL‐6, and IL‐10 in the kidney tissues were markedly elevated (Figure S3B). In comparison to WT mice, the mRNA expressions of pro‐inflammatory factors MCP‐1 and IL‐6 were decreased, whereas the anti‐inflammatory factor IL‐10 was significantly increased in *Trim65* ^−/−^ mice (Figure S3B). To evaluate renal tissue function, a GFR experiment was conducted utilizing a fluorescently labeled exogenous tracer, FITC‐sinistrin. The results demonstrated that the clearance time of the tracer in the PBS group was approximately 70 min, while the clearance time in the cisplatin‐induced WT mice group was significantly extended. In contrast, the tracer clearance time in *Trim65* ^−/−^ mice was significantly shorter than that of WT mice (Figure S2E,F). Furthermore, *Trim65* ^−/−^ mice exhibited greater tolerance to cisplatin‐induced death than WT mice (Figure S2G). These findings indicate that TRIM65 deficiency protects against AKI in experimental mice.

### 2.3 TRIM65 Physically Binds to VDAC1

**FIGURE 3:**
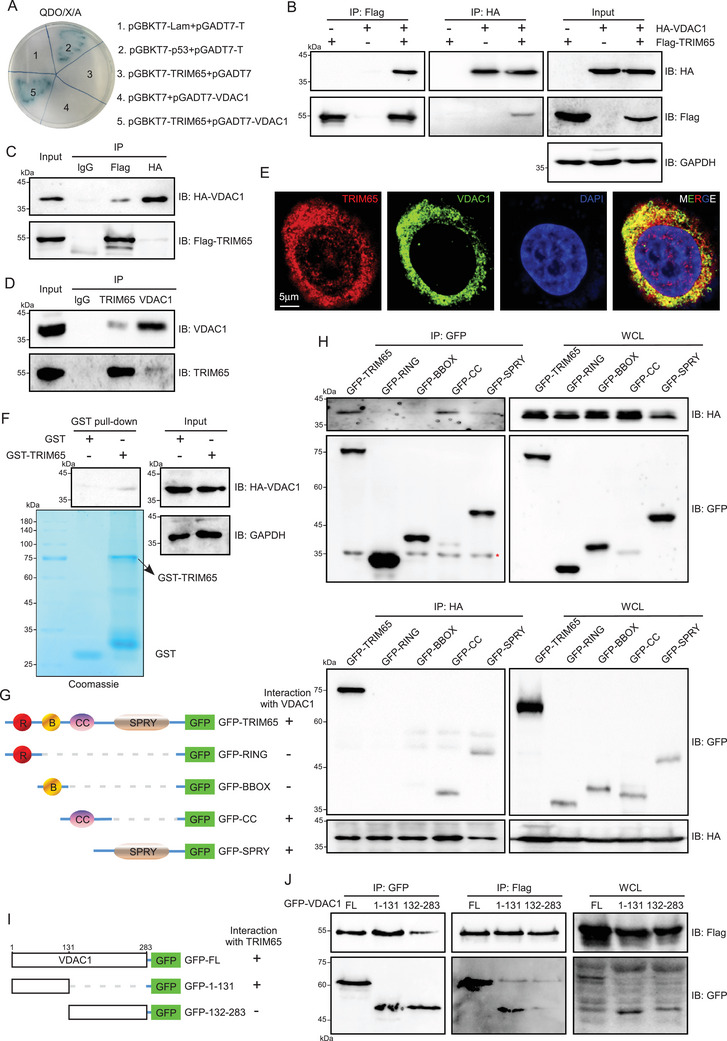
TRIM65 physically binds with VDAC1. (A) The interaction between TRIM65 and VDAC1 was validated through the usage of a yeast two‐hybrid system. (B) The interaction between TRIM65 and VDAC1 was confirmed by Co‐IP and western blot analysis. (C) Co‐IP and western blot analysis were conducted on lysates of HEK293T cells co‐transfected with HA‐VDAC1 and Flag‐TRIM65, with IgG serving as a negative control. (D) Co‐IP and western blot analysis were conducted on lysates of HK‐2 cells, with IgG serving as a negative control. (E) The co‐localization and distribution of TRIM65 and VDAC1 in endogenous HK‐2 cells were detected by immunofluorescence. Untreated HK‐2 cells were fixed with 4% paraformaldehyde and incubated with mouse anti‐TRIM65 and rabbit anti‐VDAC1 antibodies overnight. The TRIM65 protein was labeled with CoraLite594, while the VDAC1 protein was labeled with CoraLite488. A representative confocal microscopy image was acquired using a 63× oil objective. (F) Purified GST and GST‐TRIM65 fusion protein were incubated with cell lysates of HEK293T cells transfected with 10 µg HA‐VDAC1 plasmid. Following the GST pull‐down, the eluted protein and cell lysates were subjected to western blot analysis using the specified antibodies. The purified proteins of GST and GST‐TRIM65 were analyzed by Coomassie brilliant blue staining. (G) Schematic representation of the generation of serial cDNAs of truncation mutants for TRIM65 fused with pEGFP‐C1 by subcloning technique. (H) Co‐IP and western blot analysis were employed to investigate the interaction of TRIM65 and its truncation mutants with VDAC1 in the lysates of HEK293T cells co‐transfected with HA‐VDAC1, Flag‐TRIM65 (full length), and its truncation mutants. (I) Schematic of generation of serial cDNAs of deletion mutants for VDAC1 fused with pEGFP‐C1. (J) Co‐IP and western blot analysis of the interaction of VDAC1 and its deletion mutants with TRIM65 were conducted in the lysates of HEK293T cells co‐transfected with Flag‐TRIM65, GFP‐VDAC1 full length, and its deletion mutants.

To elucidate the mechanism of action of TRIM65 in AKI, we employed yeast two‐hybridization to identify potential substrates [14]. Among these, VDAC1 emerged as a particularly promising candidate, given that VDAC1 has recently been reported to play a pivotal role in AKI [16, 17]. First, a yeast two‐hybrid system was employed to ascertain the interaction between TRIM65 and VDAC1 (Figure 3A). To further substantiate these findings, a co‐immunoprecipitation (co‐IP) experiment was conducted in HEK293T cells. As shown in Figure 3B,C, TRIM65 can bind with VDAC1 and vice versa. Furthermore, the interaction between the two entities was confirmed in HK‐2 cells (Figure 3D). Another TRIM family protein, TRIM47, which is in the adjacent position of TRIM65 in human chromosome 17, was observed to fail to bind VDAC1 (Figure S4A). In contrast, Bax2, a known VDAC1 binding protein, was found to detect their interaction in HK‐2 cells (Figure S4B). Given the interaction between TRIM65 and VDAC1, we sought to determine whether they co‐localize at the subcellular level in HK‐2 cells. Immunofluorescence experiments demonstrated that TRIM65 was predominantly cytoplasmic, with partial nuclear expression, while VDAC1 was exclusively cytoplasmic and exhibited strong co‐localization with TRIM65 (Figure 3E). To ascertain whether there is a physical direct interaction between TRIM65 and VDAC1, a GST pull‐down experiment was conducted. The results showed that GST‐TRIM65 could pull down VDAC1 in vitro, whereas none of the VDAC1 protein interacted with the beads coated with GST (Figure 3F), which suggests that TRIM65 may directly interact with VDAC1.

TRIM65, a member of the TRIM protein family, is composed of four domains: the Really Interesting New Gene (RING), B‐Box‐type Zinc Finger (BBOX), Coiled Coil (CC), and SPla domain of the Ryanodine Receptor (SPRY) [14]. To identify which domain of TRIM65 was responsible for the interaction with VDAC1, plasmid constructs were generated, encoding each domain, to perform a co‐IP assay (Figure 3G,H). The results revealed that the CC and SPRY domain of TRIM65 were indispensable for the interaction with VDAC1 (Figure 3G,H). Conversely, our findings indicated that the amino acids sequence 1–131 of VDAC1, rather than the amino acids sequence 132–283, was sufficient for binding to TRIM65 (Figure 3I,J).

### 2.4 TRIM65 Mediates Ubiquitination of VDAC1

**FIGURE 4:**
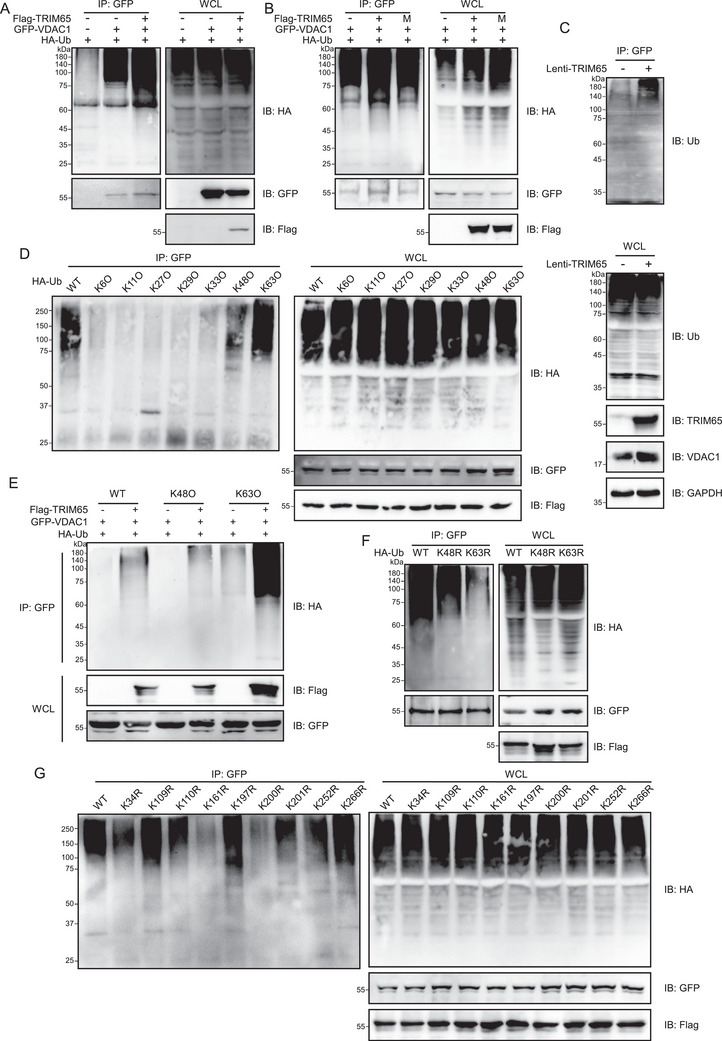
TRIM65 mediates ubiquitination of VDAC1. (A) The impact of TRIM65 on VDAC1 ubiquitination was discerned through a Co‐IP experiment and subsequent western blot analysis. (B) The ubiquitination of VDAC1 was detected in HEK293T cells that had been transfected with plasmids expressing GFP‐VDAC1, Flag‐TRIM65 (E3 ligase‐dead mutant), and HA‐Ub. Following a 48‐h incubation period, the cell lysates were subjected to Co‐IP and immunoblot analysis using the specified antibodies. (C) The ubiquitination of VDAC1 was detected in HK‐2 cells. (D) HEK293T cells were co‐transfected with Flag‐TRIM65, GFP‐VDAC1, and HA‐Ub (WT) or its mutant plasmids (HA‐Ub‐K6O, HA‐Ub‐K11O, HA‐Ub‐K27O, HA‐Ub‐K29O, HA‐Ub‐K33O, HA‐Ub‐K48O, and HA‐Ub‐K63O). At 48 h post standard calcium phosphate transfection, proteins were immunoprecipitated with anti‐GFP tag antibodies and subsequently analyzed by immunoblot analysis with the indicated antibodies. (E) HEK293T cells were co‐transfected with GFP‐VDAC1 and Flag‐TRIM65 plus HA‐Ub (WT), HA‐Ub‐K48, or HA‐Ub‐K63. Following a 48‐h incubation period, the cell lysates were subjected to Co‐IP and immunoblot analysis using the specified antibodies. (F) HEK293T cells were co‐transfected with GFP‐VDAC1 and Flag‐TRIM65 plus HA‐Ub, HA‐Ub‐K63R, or HA‐Ub ‐K48R. Following a 48‐h incubation period, the cell lysates were subjected to Co‐IP and immunoblot analysis using the specified antibodies. (G) The polyubiquitination sites of VDAC1 by TRIM65 were identified in the lysates of HEK293T cells co‐transfected with GFP‐VDAC1 or its point mutation plasmids, in which lysine (K) residues were substituted with arginine (R), Flag‐TRIM65, and HA‐Ub (WT) for 48 h. Coimmunoprecipitation and immunoblot analysis were performed with the indicated antibodies.

Given that TRIM65 has been demonstrated to possess E3 ubiquitin ligase activity, the aim of this study was to investigate the impact of TRIM65 on VDAC1 ubiquitination. As shown in Figure 4A,B, TRIM65 markedly augmented the ubiquitination of VDAC1, whereas the enzymatically inactive mutant of TRIM65 did not result in an increase in the ubiquitination modification of VDAC1. Additionally, the overexpression of TRIM65 was observed to markedly elevate the ubiquitination level of VDAC1 in HK‐2 cells (Figure 4C). It has been demonstrated that E3 ubiquitin ligases can mediate diverse types of ubiquitin chain modification to the substrate, thereby exerting distinct functions [18]. To determine the type of ubiquitin chains added by TRIM65 to VDAC1, a series of ubiquitin mutants were utilized in ubiquitination assays. The results demonstrated that both K48‐ and K63‐linked ubiquitin modifications of VDAC1 appeared in the presence of TRIM65, while other types of ubiquitin modifications were not detected (Figure 4D). As illustrated in Figure 4E, the overexpression of TRIM65 resulted in a notable elevation in the K48‐ and K63‐linked ubiquitination of VDAC1. Moreover, ubiquitination experiments were conducted using ubiquitin molecules that had been mutated from lysine to arginine at positions 48 and 63 (K48R and K63R), thereby confirming that these two lysine residues are pivotal sites on VDAC1 polyubiquitination modified ubiquitin (Figure 4F). Therefore, TRIM65‐conjugated ubiquitin chains on VDAC1 were predominantly K48 and K63 ubiquitin linkages. This was evidenced by the efficient conjugation of ubiquitin containing K48 and K63 as the sole lysine residue, whereas ubiquitin with K48R or K63R mutation was not.

To identify the precise site of ubiquitination by TRIM65, we generated VDAC1 mutants bearing single lysine‐to‐arginine substitutions at each potential ubiquitylation site. As illustrated in Figure 4G, the extent of ubiquitin conjugation to the VDAC1 mutant with lysine 161 to arginine (K161R) and lysine 200 to arginine (K200R) was substantially diminished in comparison to that of either the WT VDAC1 or any mutant bearing single lysine to arginine substitutions on each of the other ubiquitylation sites. These findings suggest that lysine 161 and 200 are the primary sites for TRIM65‐mediated VDAC1 ubiquitination.

### 2.5 TRIM65 Maintains VDAC1 Protein Stability

**FIGURE 5:**
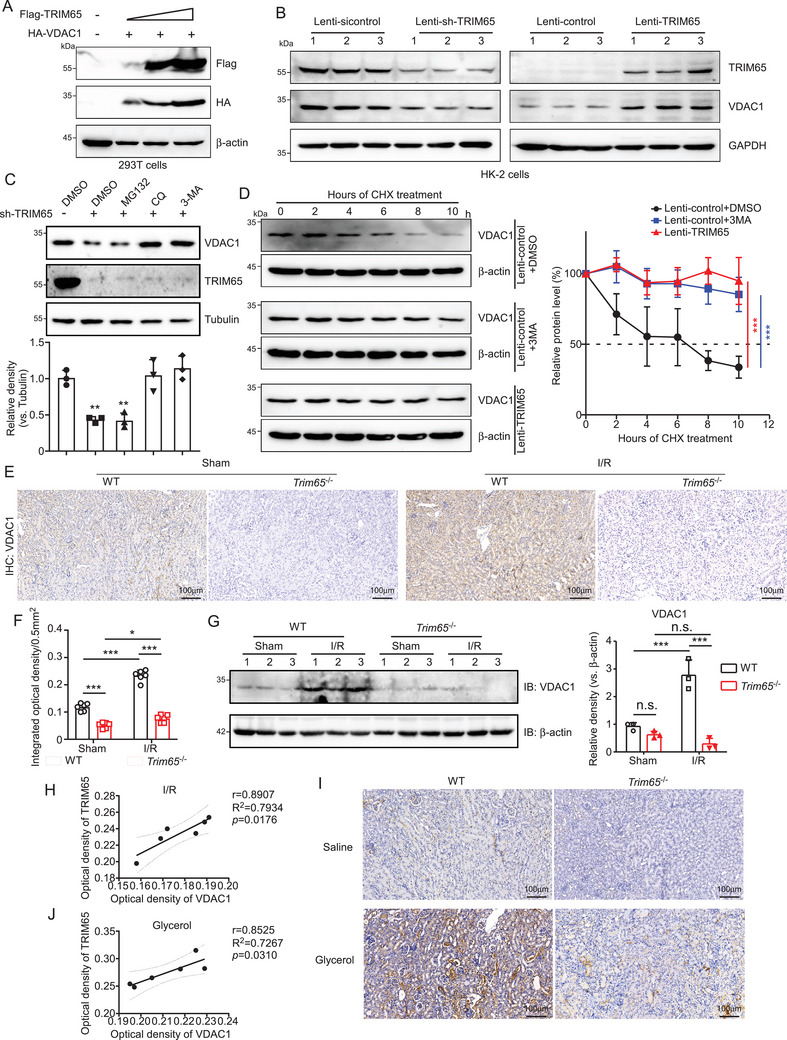
TRIM65 maintains VDAC1 protein stability. (A) The impact of TRIM65 protein overexpression on the VDAC1 protein level. HEK293T cells were co‐transfected with HA‐VDAC1 and varying concentrations of Flag‐TRIM65, which were increased in a stepwise manner. Following a 48‐h incubation period, cell lysates were subjected to immunoblot analysis using Flag and HA antibodies. β‐Actin was employed as a standardization control. (B) Immunoblotting was employed to assess the impact of TRIM65 overexpression and knock‐down on VDAC1 stability following HK‐2 cell infection with lentivirus for 72 h. (C) The impact of inhibitors on TRIM65‐mediated downregulation of VDAC1 was also investigated. The protein levels of VDAC1 and TRIM65 in HK‐2 cells treated with 20 µM MG132, 100 µM CQ, and 10 mM 3‐MA, respectively, for 24 h were analyzed by immunoblotting with an anti‐VDAC1 antibody. The inhibitors were prepared in DMSO. The relative level of VDAC‐1 protein was normalized to that of tubulin. (D) HK‐2 cells expressing Lenti‐control and Lenti‐TRIM65 were treated with 100 µM CHX plus DMSO or 3‐MA for the indicated times. The expression of VDAC1 was detected in western blots and normalized to β‐actin. The data are presented as the mean ± SD. (E) Representative IHC staining of VDAC1 in mouse kidneys treated with ischemia/reperfusion or sham surgery in four groups of mice. Scale bar: 100 µm. (F) The optical density of the VDAC1 IHC score. (G) Western blot analysis of VDAC1 was conducted on kidney tissue homogenates following I/R injury, with quantification of the VDAC1 western blot band performed using ImageJ software. (H) Correlation analysis based on IHC optical density values of TRIM65 and VDAC1 in kidney tissue from mice with I/R‐induced AKI. (I) Representative IHC staining detection of VDAC1 in mouse kidneys treated with saline or glycerol injection in four groups of mice. Scale bar: 100 µm. (J) Correlation analysis based on IHC optical density values of TRIM65 and VDAC1 in kidney tissue from mice with glycerol‐induced AKI. All data were subjected to the two‐way ANOVA and Tukey's post hoc test were used for multiple group comparisons. Statistical significance was indicated by *p < 0.05, ***p < 0.001, n.s. indicates no significance. All experiments were repeated three times.

Given that protein ubiquitination affects a multitude of cellular processes by regulating protein degradation and protein‐protein interactions [19], we proceeded to determine the effect of TRIM65 on VDAC1 stability. The results showed that TRIM65 increased VDAC1 expression concentration dependently (Figure 5A). However, the overexpression of TRIM65 did not result in an increase in protein level following the mutation of the site where TRIM65 ubiquitinates VDAC1 (Figure S5). In HK‐2 cells, the knockdown of TRIM65 was observed to significantly reduce VDAC1 protein levels and vice versa (Figure 5B). Polyubiquitin linkages via lysine 48 (K48) or 63 (K63) can result in the differential targeting of proteins for 26S proteasomal degradation or endosome trafficking to the lysosome [20]. To investigate how TRIM65 regulates VDAC1 protein levels in renal cells, it was necessary to determine which of the major VDAC1 degradation pathways was affected by TRIM65. Inhibitors for autophagosome (3‐methyladenine [3‐MA]), lysosome (chloroquine [CQ]), and proteasome (MG132) were administered to control or TRIM65 knockdown HK‐2 cell; both CQ and 3‐MA abolished the negative regulation of sh‐TRIM65 on VDAC1 levels (Figure 5C), indicating that TRIM65 blocks the degradation of VDAC1 through the autophagy pathway. The impact of TRIM65 on the stability of VDAC1 was subsequently verified. A cycloheximide chase assay demonstrated that the overexpression of TRIM65 and 3‐MA markedly impeded the degradation of VDAC1 protein in HK‐2 cells (*p* < 0.001, Figure 5D). IHC and western blot analysis of renal tissues from I/R mice revealed that VDAC1 expression was increased in AKI, whereas it was lower in TRIM65 knockout mice in both sham‐operated and I/R groups (Figure 5E–G). The correlation analysis revealed that the protein level of VDAC1 in renal tissue exhibited a significant positive correlation with the expression of TRIM65 in the I/R‐induced AKI mouse model (*p* < 0.05, *r* = 0.8907, Figure 5H). Further validation in the rhabdomyolysis‐induced AKI model demonstrated that the expression of VDAC1 was consistent with that of TRIM65 (Figure 5I). Additionally, correlation analysis revealed a significant positive correlation between the expression of TRIM65 and VDAC1 (*p* < 0.05, *r* = 0.8525, Figure 5J). In conclusion, TRIM65 plays a role in stabilizing VDAC1 and regulating its expression in AKI.

### 2.6 TRIM65 Aggravated Mitochondrial Dysfunction

**FIGURE 6:**
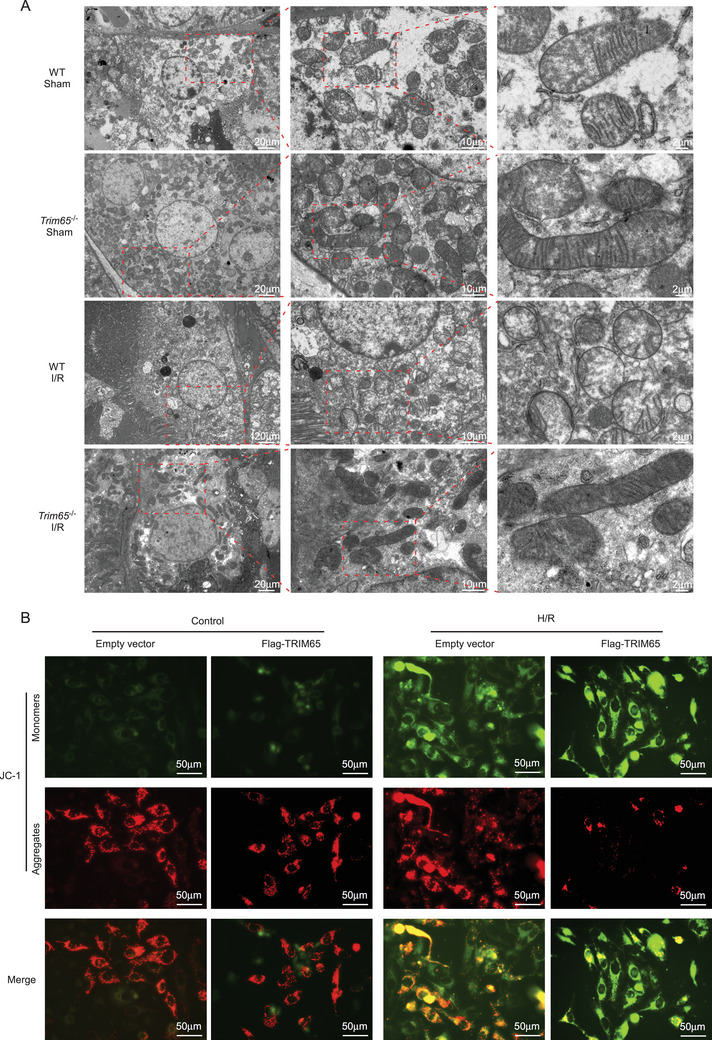
TRIM65 aggravated mitochondrial dysfunction. (A) TEM was employed to observe the morphological changes of mitochondria in the kidney tissues of mice that had undergone either renal I/R or sham surgery for 24 h. The scale bars are presented as 20, 10, and 2, respectively. (B) The effects of mitochondrial membrane potential in HK‐2 cells expressing Lenti‐control or Lenti‐TRIM65 under H/R injury or normoxia were observed. Scale bars are presented as 50 µm.

Given that TRIM65 may interact with the mitochondrial outer membrane protein VDAC1 to affect I/R‐induced AKI, we postulated that TRIM65 may exert a certain impact on mitochondrial function. Transmission electron microscopy (TEM) was employed to ascertain whether TRIM65 affects the ultrastructure of mitochondria in AKI. As illustrated in Figure 6A, the mitochondrial cristae are well developed in normal renal cells, with the cristae connected to the inner membrane. Furthermore, TRIM65 knockdown does not appear to affect mitochondrial structure under normal conditions. However, this organization was lost during the progression of AKI, and the mitochondrial cristae were fewer in number and unorganized in AKI renal tissue cells. Additionally, there was a notable presence of swollen vacuoles and enlarged individual organelles, which did not appear to be connected in a mitochondrial network (Figure 6A). TRIM65 deletion ameliorated the disruption and damage to mitochondrial structure in AKI renal cells (Figure S6A). These findings indicate that AKI results in a reduction in the structural integrity of mitochondria, and that TRIM65 knockdown protects against AKI by preventing the destruction of mitochondrial ultrastructure. It is well established that the mitochondrial transmembrane potential (ΔΨm) serves as a crucial indicator of mitochondrial function [21]. A decline in this parameter is frequently considered an indicator of mitochondrial dysfunction [21]. JC‐1 is a ratiometric probe that monitors ΔΨm by determining the relative dual emission of the mitochondrial J aggregates (red fluorescent) versus JC‐1 monomers (green fluorescent). In normal cells, JC‐1 is predominantly located in the mitochondria, resulting in the accumulation of red J aggregates with a slight green fluorescent monomeric form remaining in the cytoplasm (Figure 6B). In contrast, following hypoxia‐reoxygenation treatment, the green fluorescence of J monomers in the cytoplasm of HK‐2 cells gradually increased, while the red fluorescence of J aggregation proteins (normal mitochondria) was reduced, indicating a collapse of ΔΨm (Figure 6B). This indicated that the mitochondria had been severely damaged. However, overexpression of TRIM65 significantly inhibited J aggregates (Figure S6B), suggesting that TRIM65 may act as a negative regulator of mitochondrial function in AKI.

### 2.7 Protective Effect of TRIM65 Deficiency in AKI Reversed by VDAC1 Overexpression

**FIGURE 7:**
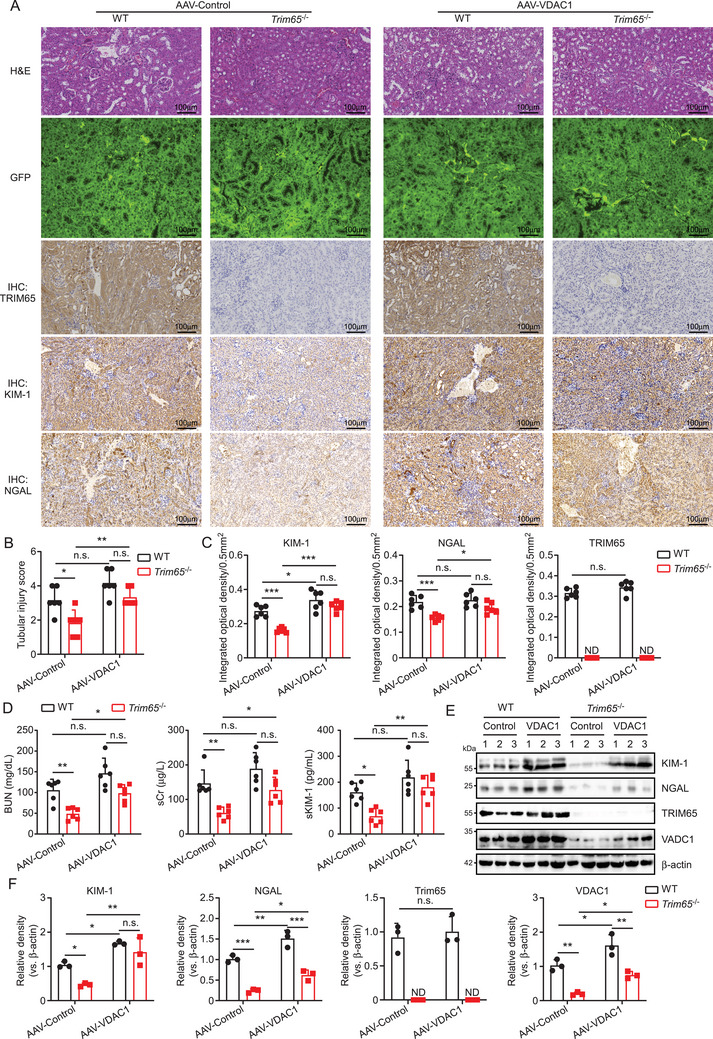
VDAC1 is a pivotal target of TRIM65 in regulating I/R‐induced AKI. (A) Representative images of green fluorescence and IHC staining of renal tissue from mice pathological sections. The scale bars are presented as 100 µm. (B) The renal tubular injury score. (C) The degree of staining and the positive range of TRIM65, KIM‐1, and NGAL are evaluated under an optical microscope, and the IHC is scored accordingly. The study was conducted with *n* = 6 mice per group. (D) The levels of serum BUN, creatinine, and KIM‐1 in the peripheral blood of four groups of C57BL/6 mice (AAV‐VDAC1+WT, AAV‐VDAC1+*Trim65* ^−/−^, AAV‐control+WT, AAV‐control+ *Trim65* ^−/−^) were analyzed. (E) Immunoblot analysis was employed to quantify the levels of KIM‐1, NGAL, TRIM65, and VDAC1 protein expression in mice subjected to renal I/R in the four aforementioned groups. β‐Actin was utilized as an internal control. (F) The quantification of KIM‐1, NGAL, TRIM65, and VDAC1 western blot bands in mice was conducted using ImageJ software. *p < 0.05, **p < 0.01, ***p < 0.001, n.s. indicates no significance.

To ascertain the role of TRIM65 in AKI by targeting VDAC1, a serotype 9 adeno‐associated virus vector packaged in a serotype 9 capsid (AAV2/9) was employed. This vector has been demonstrated to exhibit excellent renal tissue specificity and efficacy in transducing the carried gene [22]. In brief, AAV2/9 constructs carrying either the VDAC1 gene or EGFP were injected into renal tissues via intrapelvic injection in WT and *Trim65* knockout mice. Twenty‐one days later, I/R‐induced AKI was performed, and high green fluorescence in renal tissues of all groups was observed by fluorescence microscopy. This suggests that AAV2/9 successfully mediated either VDAC1 or EGFP into the kidneys (Figure 7A). The degree of AKI in WT and *Trim65* ^−/−^ mice induced by I/R was not altered by the empty AAV vector. In contrast, TRIM65 knockdown showed a significant protective effect (Figure 7A,B). However, renal overexpression of VDAC1 almost completely abolished the protective effect of TRIM65 deletion and also evidently exacerbated AKI in WT mice (Figure 7A,B). It is noteworthy that VDAC1 did not affect the expression of TRIM65 in renal tissues of WT mice (Figure 7A,C). Consequently, serum levels of BUN, sCr, and Kim‐1 were markedly diminished in AAV‐control‐treated *Trim65* ^−/−^ mice in comparison to AAV‐control‐treated WT mice. However, AAV‐VDAC1 treatment yielded no statistical difference between the two groups in WT and *Trim65* ^−/−^ AKI mice (Figure 7D). Western blot analysis confirmed that the injection of AAV‐VDAC1 resulted in the overexpression of VDAC1 in the kidney tissues of both WT mice and *Trim65* knockout mice (Figure 7E,F). The VDAC1 significantly increased the protein levels of KIM‐1 and NGAL in the kidney tissues of *Trim65* ^−/−^ AKI mice (Figure 7A,C,E,F). Indeed, the overexpression of VDAC1 was observed to markedly elevate the protein levels of KIM‐1 and NGAL in the kidney tissues of WT AKI mice (Figure 7A,C,E,F). Collectively, the overexpression of VDAC1 in kidney tissues effectively counteracted the protective effect of TRIM65 knockdown on AKI, thereby suggesting that TRIM65 may potentially target VDAC1 to regulate AKI.

## 3 Discussion

Ubiquitination is a highly conserved biological process that is dependent on E1 ubiquitin‐activating enzymes, E2 ubiquitin‐conjugating enzymes, and E3 ubiquitin ligases [20]. Among these, E3 ubiquitin ligases have been identified as having excellent substrate recognition capabilities and are therefore considered attractive potential drug targets [23]. The TRIM protein family, a class of E3 ligases with RING finger structural domains, is emerging as a key player in the field of inflammation research [24]. A previous study has demonstrated that TRIM72 plays a role in facilitating the repair of renal proximal tubular epithelium cells, thereby protecting against the development of AKI [25]. Sun et al. [26] recently reported that TRIM21 promotes ferroptosis through the ubiquitination and degradation of GPX4, thereby exacerbating I/R‐induced AKI. Inhibition of the TLR4/NF‐κB signaling pathway by TRIM27 exerts anti‐inflammatory and anti‐apoptotic effects, thereby effectively alleviating LPS‐induced HK‐2 cell damage and AKI [27]. However, the role of the TRIM protein family in AKI remains poorly understood.

To address this issue, we conducted a study to examine changes in the mRNA levels of the entire TRIM family in two AKI mouse models. The findings revealed that a few TRIM protein family members exhibited notable alterations in rhabdomyolysis and I/R‐induced AKI. Of these, *Trim65* exhibited one of the most pronounced changes. TRIM65 expression was predominantly observed in renal tubular epithelial cells, with a notable concentration in the cytoplasm and a relatively minor presence in the nucleus. There was a notable increase in the expression of this protein in the context of AKI. In contrast, the protein levels of TRIM65 were observed to be lower in glomeruli, which did not undergo significant changes in AKI. Bioinformatics studies have indicated that the transcription factor HMGA1 is predicted to be an upstream regulatory factor of TRIM65. Luciferase reporter gene assays have confirmed that TRIM65 is positively transcriptionally regulated by HMGA1 [28]. Additionally, TRIM65 has been identified as a potential target for miR‐515‐5p [29]. Recent studies have demonstrated that the expression of TRIM65 in macrophages can be inhibited by LPS through the ERK1/2 pathway, which can be blocked by the ERK1/2 inhibitor U0126 [30]. Furthermore, *Trim65* knockout has been demonstrated to facilitate macrophage activation by activating the ERK1/2 signaling pathway, thereby establishing a positive feedback loop [30]. These findings imply that the elevation of TRIM65 in AKI may be regulated by transcription factors or miRNAs, in addition to other upstream effector factors that have yet to be identified.

Our previous studies have demonstrated that TRIM65 attenuates endothelial inflammation and acute lung injury by ubiquitination and degradation of VCAM‐1 [10]. Furthermore, our recent findings have revealed that TRIM65 plays a pivotal role in intestinal I/R injury by targeting TOX4 to inhibit apoptosis [14]. It is also noteworthy that our findings indicate that TRIM65 plays a role in the development of renal cell carcinoma by regulating BTG3 and the cell cycle [31]. Moreover, the TRIM65 protein plays a role in the regulation of renal fibrosis by targeting the NUDT21‐mediated selective polyadenylation pathway [32]. However, the role of TRIM65 in other acute inflammatory conditions, including AKI, has yet to be elucidated. The present study indicates that TRIM65 deficiency significantly attenuates AKI in multiple models, including rhabdomyolysis, I/R, and cisplatin‐induced AKI models. Our findings suggest that TRIM65 is a highly promising drug target for AKI.

A growing body of evidence indicates that mitochondrial dysfunction is a pivotal factor in the pathogenesis of AKI [32]. Mitochondrial pathology manifests prior to the onset of detectable renal dysfunction and persists in renal tubular epithelial cells that fail to recover following AKI [32]. It is noteworthy that the protection of mitochondria prior to the onset of AKI has been demonstrated to prevent the development of AKI and to attenuate the transition from AKI to chronic kidney disease (CKD) [33]. As anticipated, the I/R injury resulted in the formation of mitochondrial ultrastructural abnormalities, loss of mitochondrial cristae, and swelling in the kidney. Intriguingly, deletion of TRIM65 significantly alleviated AKI‐induced mitochondrial disruption. Furthermore, overexpression of TRIM65 aggravated H/R‐induced mitochondrial membrane potential abnormalities. It is noteworthy that TRIM65 did not affect mitochondrial structure under normal physiological conditions. Indeed, TRIM65 was observed to co‐localized with the mitochondrial outer membrane protein VDAC1. Moreover, TRIM65 was able to directly bind with and maintain the stability of VDAC1. VDAC1 is highly conserved in mammals and plays a crucial role in maintaining mitochondrial structure and function [34]. It has been demonstrated that the overexpression of VDAC1 results in mitochondrial dysfunction and the subsequent induction of cell death. Conversely, the inhibition of VDAC1 has been shown to effectively suppress intracellular oxidative stress and restore mitochondrial function [35]. It is hypothesized that TRIM65 deficiency results in the destabilization of VDAC1, thereby protecting against mitochondrial structural and functional damage in AKI and ultimately attenuating AKI. The reduction of TRIM65 expression or the inhibition of its function may represent a novel strategy for the prevention and treatment of AKI.

It is worthy of note that TRIM65 is capable of mediating both K48‐linked and K63‐linked ubiquitination modifications of VDAC1. Previous studies have indicated that K48‐linked ubiquitination modifications are involved in proteasomal degradation, whereas K63‐linked ubiquitination mediates protein–protein interactions or substrate stability [20]. The findings of this study indicate that K48/K63‐linked ubiquitination of VDAC1 is associated with enhanced protein stability. Notably, Tang and colleagues previously demonstrated that TRIM65 facilitates the K48/K63‐linked ubiquitination of NLRP3, thereby inhibiting NLRP3 inflammasome assembly, caspase‐1 activation, and IL‐1β secretion [12]. In addition, Wang and colleagues [36] have recently discovered that the E3 ubiquitin ligase TRAF6 is responsible for the non‐proteolytic K48/K63‐linked ubiquitination of ATG9A, enhancing its binding to Beclin1 and promoting autophagy in response to oxidative stress. However, Chen et al. 37]. demonstrated that the E3 ligase MID1 induces IFNAR2 to undergo both K48‐ and K63‐linked polyubiquitination, which promotes its degradation by lysosome‐dependent pathways. This suggests that the K48/K63‐linked ubiquitination is a multifaceted process, and the precise functions and molecular mechanisms warrant further investigation. It has been demonstrated that VDAC1 can be mono‐ or polyubiquitinated by the E3 ubiquitin ligase Parkin in a PINK1‐dependent manner, which plays an important role in the pathogenesis of Parkinson's disease [38, 39] and liver fibrosis [40]. The present study identifies a novel ubiquitin ligand protein of VDAC1 and mediates ubiquitination of the K48/K63 linkage at a different site from that previously reported. This provides new insights that will further elucidate the regulatory mechanism of VDAC1.

VDAC1 is a multifunctional protein that plays a key role in cellular metabolism, intracellular calcium homeostasis, oxidative stress, and mitochondria‐mediated apoptosis [34]. In a study by Nowak et al. [17], it was found that the global deletion of VDAC1 hinders morphological recovery and the improvement of renal function in the proximal tubules of I/R‐injured mice. This prevents the repair of mitochondrial damage, thereby increasing the level of AKI and renal fibrosis. However, renal tubule‐specific knockout of *Vdac1* in mice demonstrated a notable reduction in mitochondrial damage induced by several AKI models, and inhibition of renal tubular VDAC1 was observed to offer significant protection against AKI [16]. The present study demonstrated that *Trim65* knockout resulted in a significant reduction of VDAC1 and attenuation of mitochondrial damage. Furthermore, overexpression of VDAC1 in renal tissues mediated by AAV vectors was resistant to the protective effect of TRIM65 deficiency on AKI. These findings suggest that targeted inhibition of renal tubular VDAC1 can effectively attenuate AKI. Nevertheless, the precise mechanism by which TRIM65‐targeted VDAC1 exerts its effects on mitochondrial injury in AKI remains to be elucidated, thereby warranting further investigation in future studies.

Furthermore, it is essential to recognize the potential constraints of the study, including the possibility of discrepancies between mouse models and human physiology. It is acknowledged that several experimental models are only able to partially replicate the development of human disease, a fact that is particularly evident in the case of AKI. Although the experimental models currently used to study human AKI have limitations, they remain valuable tools for advancing our understanding of AKI and developing targeted therapeutic strategies. Additionally, the systemic knockout *Trim65* mice employed in this study do not exclude the influence of non‐renal epithelial cells. To further elucidate the potential influence of other cells, such as macrophages and endothelial cells, on AKI, we are also constructing conditional knockout *Trim65* mice.

## 4 Conclusions

In conclusion, the expression of TRIM65 is increased in cases of AKI, which results in mitochondrial structural and functional abnormalities. The TRIM65 protein has been demonstrated to positively regulate AKI by mediating K48/K63‐linked ubiquitination modifications of VDAC1 at the K161 and K200 sites. Conversely, TRIM65 deficiency has been observed to facilitate the autophagic degradation of VDAC1, thereby alleviating the imbalance in mitochondrial function and attenuating AKI. It may be postulated that targeting the mitochondrial function regulated by TRIM65/VDAC1 represents a potential strategy for the treatment of AKI.

## 5 Materials and Methods

### 5.1 Animals

Female C57BL/6 WT mice (20–25 g) were obtained from Hunan Shrek Jingda Experimental Animal Co., Ltd. The *Trim65* ^−/−^ mice were generated in the C57BL/6 background by backcrossing the C57BL/6 WT mice with *Trim65* ^+/−^ mice (Cyagen Biosciences, Inc.) as previously described [14, 15]. The genotype was identified by polymerase chain reaction (PCR). The mice were housed at a temperature of 22 ± 2°C, with a 12‐h light/dark cycle and 50% humidity, and were provided with food and water ad libitum. All animal protocols employed in this study were approved by the Institutional Animal Care and Use Committee of the First Affiliated Hospital of Nanchang University (approval number: CDYFY‐IACUC‐202301QR010). Eight‐week‐old female mice were randomly allocated to each group of six, with 12 mice in each group, for the survival experiment. The number of animals used and the associated suffering were kept to a minimum throughout the study.

### 5.2 Rhabdomyolysis‐Induced AKI Model

Following an overnight fast, all animals were anaesthetized using isoflurane (2%) and oxygen flow (2 L/min) to induce and maintain anesthesia. Once the mice had reached the appropriate depth of anesthesia, they were weighed and then intramuscularly injected with 50% glycerol (8 mL/kg, with a solvent of normal saline) into the hind limbs. The mice in the control group were weighed and injected with an equal volume of normal saline into the muscles. Observation of the hind limbs of mice that had just been injected with 50% glycerol revealed that they were in a straight and dragging state. Euthanasia was performed 24 h later, after which eyeball blood samples and kidney tissue samples were collected.

### 5.3 Ischemia/Reperfusion Induced‐AKI Model

The mice were fasted overnight prior to anesthesia with 2% pentobarbital sodium (70 µg/kg). The mice were positioned in a supine position on the preheating pad. A 1‐ to 2‐cm incision was made at the abdominal midline, following the requisite sterilization and skin preparation. Subsequently, the intestine was extracted with the aid of two cotton swabs, thereby preventing damage to the surrounding blood vessels and nerves. Subsequently, the renal pedicle was exposed. The kidney artery and vein were then separated and clamped with precision using a non‐traumatic microsurgical vascular clip. The color of the kidney altered from bright red to dark red, indicating successful ischemia. Following a period of 40 min, the clip was released with great care to prevent the rupture and subsequent bleeding of the renal pedicle. The color of the kidney changed from dark red to bright red, indicating successful reperfusion. The sham group returned the kidney and intestine to the abdominal cavity after surgical exploration and kidney exposure without clipping the renal pedicle. The incision was then closed with 4/0 silk thread layer by layer. After 24 h, the mice were anesthetized and euthanized with isoflurane (2%) for the collection of eyeball blood samples and kidney tissue samples.

### 5.4 H&E and IHC

The slides derived mouse kidney tissues were immersed in xylene for 20 min. Subsequently, the slices were incubated in ethanol with decreasing concentration gradients for a period of 5 min, followed by a further 5 min in double‐distilled water and PBS. The H&E solution was added in a stepwise manner. The slices were dehydrated in ethanol with increasing concentration gradients and placed in xylene. After sealing with neutral gum, renal tubular damage images were acquired with a pathological section scanner (Sunny Optical Technology 193 (Group) Company Limited, China) in a blinded manner by a semiquantitative method as previously described [41]. The images were scored in accordance with the following criteria: A score of 0 was indicative of normal tissue, while a score of 1 indicated minor damage, defined as less than 5% of the tissue affected, A score of 2 was represented as mild damage, occurring in tissue with 5%–25% damage, while a score of 3 signified moderate damage, affecting tissue with 25%–75% damage. Finally, a score of 4 was assigned to cases of severe damage, occurring in tissue with more than 75% damage. The IHC procedure was conducted by the previously described methodology [14]. The primary antibodies employed in this study included anti‐TRIM65 (Atlas Antibodies, Sweden; HPA021578, 1:400), anti‐VDAC1 (Proteintech, China; 66345‐1‐Ig, 1:50), anti‐KIM‐1 (R&D Systems, USA; AF1817, 1:400), and anti‐NGAL (R&D Systems, USA; AF1857, 1:400). The antibodies were incubated at 4°C overnight. On the following day, the slides were incubated with horseradish peroxidase (HRP)‐conjugated secondary antibodies at room temperature. Following a washing step, the positive stain was detected with 3,3’‐diaminobenzidine (DAB). The final evaluation was performed under a pathological section scanner and analyzed using the ImageScope system with a minimum of six sections.

### 5.5 Transmission Electron Microscope (TEM)

The extraction of kidney tissue from the mice was completed within 3 min of the mice being euthanized. The mouse renal cortex sample was isolated and fixed in 2.5% glutaraldehyde (Servicebio, China). The cortex tissues were embedded in resin. Ultrathin sections of 80 nm were obtained by an ultramicrotome (Leica, Germany) and examined with a TEM (HITACHI, Japan).

### 5.6 Cell Culture and Hypoxia/Reoxygenation (H/R) Procedures

The HK‐2 cells were procured from Wuhan Procell Life Science & Technology Co., Ltd. The human embryonic kidney (HEK293T) cells were obtained from the American Type Culture Collection (ATCC). All cell lines were authenticated via short tandem repeat (STR) analysis and tested for mycoplasma contamination. The cells were cultured in Dulbecco's modified Eagle's medium (DMEM) (Servicebio, China) supplemented with 10% fetal bovine serum (ExCell Bio, China) and 1% penicillin/streptomycin (NCM Biotech, China) in an incubator (Thermo Scientific, USA) set to 5% CO_2_ at 37°C. The HK‐2 cells were exposed to a well‐controlled three‐gas cell culture incubator (BioSpherix, USA) under nutrient‐deprived conditions (1% O_2_, 94% N_2_, and 5% CO_2_) for 12 h, then transferred into complete medium for 4 h of reoxygenation at 37°C and 5% CO_2_. The control group was maintained in an adequate nutrition and normoxic environment throughout the experiment.

### 5.7 JC‐1 Dye

HK‐2 cells were plated in a six‐well plate and transfected with Flag‐TRIM65 or an empty vector using Hieff Trans Liposomal Transfection Reagent (YEASEN, Shanghai, China) for 48 h. Subsequently, the cells were subjected to a H/R procedure. The cells were then rinsed with PBS and incubated with the JC‐1 fluorescent probe (Beyotime, China) at 37°C for 20 min, followed by washing twice with pre‐cooled JC‐1 staining buffer. The inverted fluorescence microscope was employed to detect the fluorescence. When the mitochondrial membrane potential was normal, JC‐1 aggregated in the mitochondrial matrix to form polymers, which exhibited red fluorescence. Conversely, when the mitochondrial membrane potential was compromised, JC‐1 was unable to aggregate in the mitochondrial matrix. Consequently, JC‐1 existed as monomers, which emitted green fluorescence. The ratio of red and green fluorescence intensity was found to be negatively correlated with early cell apoptosis.

### 5.8 Statistical Analysis

All statistical analyses were conducted using GraphPad Prism version 8.0. The data were expressed as mean ± SEM. For data that met the normality test, a Student's *t*‐test was employed for the comparison of two groups. Comparisons between multiple groups were made using one‐way ANOVA followed by Tukey's post hoc test. Comparisons between different time points were made using repeated‐measures ANOVA. Nonparametric rank‐sum tests were used for data that did not meet the normality test. A *p*‐value of less than 0.05 was considered statistically significant.

## Author Contributions

Y.L. and X.H. conceived and designed the study, prepared the figures, analyzed data, and participated in the paper writing; S.C. provided conceptual advice, performed the validation, and revised the manuscript; T.C. and Y.Z. performed the experiments, analyzed the data, and wrote the initial draft of the manuscript; L.D. completed the most of the experiments in the cisplatin‐induced AKI model. C.X., C.M., M.J., Y.H., and S.W. conducted some of the animal experiments during the study. S.W. also helped with the confocal microscopic analysis. T.X., Q. Zhu, and Q. Zhang bred and genotyped the mice, and provided assistance with some of the animal experiments. J.C. reviewed and revised the manuscript for important intellectual content, and all authors have read and approved the final manuscript.

## Conflicts of Interest

The authors declare no conflicts of interest.

## Ethics Statement

All animal procedures were approved by The Institutional Animal Care and Use Committee of The First Affiliated Hospital of Nanchang University and performed in accordance with the ARRIVE guideline (approval number: CDYFY‐IACUC‐202301QR010). All methods were carried out in accordance with relevant guidelines and regulations.

## Supporting information



Supporting Information

## Data Availability

The data analyzed during this study are included in this article. Additional supporting data are available from the corresponding authors upon reasonable request.

## References

[1] S. R. Gonsalez , A. L. Cortes , R. C. D. Silva , J. Lowe , M. C. Prieto , and L. D. Silva Lara , “Acute Kidney Injury Overview: From Basic Findings to New Prevention and Therapy Strategies,” Pharmacology & Therapeutics 200 (2019): 1–12. 30959059 10.1016/j.pharmthera.2019.04.001 PMC10134404

[2] C. Ronco , R. Bellomo , and J. A. Kellum , “Acute Kidney Injury,” Lancet 394, no. 10212 (2019): 1949–1964. 31777389 10.1016/S0140-6736(19)32563-2

[3] A. Abebe , K. Kumela , M. Belay , B. Kebede , and Y. Wobie , “Mortality and Predictors of Acute Kidney Injury in Adults: A Hospital‐Based Prospective Observational Study,” Scientific Reports 11, no. 1 (2021): 15672. 34341369 10.1038/s41598-021-94946-3 PMC8329200

[4] J. A. Kellum , P. Romagnani , G. Ashuntantang , C. Ronco , A. Zarbock , and H. J. Anders , “Acute Kidney Injury,” Nature Reviews Disease Primers 7, no. 1 (2021): 52. 10.1038/s41572-021-00284-z 34267223

[5] Q. Zhang , T. Sun , F. Yu , et al., “PAFAH2 Suppresses Synchronized Ferroptosis to Ameliorate Acute Kidney Injury,” Nature Chemical Biology 20, no. 7 (2024): 835–846. 38287154 10.1038/s41589-023-01528-7

[6] R. You , Y. Li , Y. Jiang , et al., “WWP2 Deletion Aggravates Acute Kidney Injury by Targeting CDC20/Autophagy Axis,” Journal of Advanced Research (2024). 10.1016/j.jare.2024.06.015 PMC12126736 38909885

[7] N. Zheng and N. Shabek , “Ubiquitin Ligases: Structure, Function, and Regulation,” Annual Review of Biochemistry 86 (2017): 129–157. 10.1146/annurev-biochem-060815-014922 28375744

[8] Y. Huang , Y. Xiao , X. Zhang , X. Huang , and Y. Li , “The Emerging Roles of Tripartite Motif Proteins (TRIMs) in Acute Lung Injury,” Journal of Immunology Research 2021 (2021): 1007126. 34712740 10.1155/2021/1007126 PMC8548118

[9] J. Chen , X. Feng , X. Zhou , and Y. Li , “Role of the Tripartite Motif‐Containing (TRIM) Family of Proteins in Insulin Resistance and Related Disorders,” Diabetes, Obesity & Metabolism 26, no. 1 (2024): 3–15. 10.1111/dom.15294 37726973

[10] Y. Li , X. Huang , F. Guo , et al., “TRIM65 E3 Ligase Targets VCAM‐1 Degradation to Limit LPS‐Induced Lung Inflammation,” Journal of Molecular Cell Biology 12, no. 3 (2020): 190–201. 31310649 10.1093/jmcb/mjz077 PMC7181722

[11] X. F. Ma , Y. R. Zhou , Z. X. Zhou , et al., “TRIM65 Suppresses oxLDL‐Induced Endothelial Inflammation by Interaction With VCAM‐1 in Atherogenesis,” Current Medicinal Chemistry 31, no. 30 (2024): 4898–4911. 37608612 10.2174/0929867331666230822152350

[12] T. Tang , L. i. P. X. Zhou , et al., “The E3 Ubiquitin Ligase TRIM65 Negatively Regulates Inflammasome Activation Through Promoting Ubiquitination of NLRP3,” Frontiers in Immunology 12 (2021): 741839. 34512673 10.3389/fimmu.2021.741839 PMC8427430

[13] J. Luo , Y. Luo , J. Chen , et al., “Intestinal Metabolite UroB Alleviates Cerebral Ischemia/Reperfusion Injury by Promoting Competition Between TRIM65 and TXNIP for Binding to NLRP3 Inflammasome in Response to Neuroinflammation,” Biochimica et Biophysica Acta (BBA) – Molecular Basis of Disease 1870, no. 4 (2024): 167056. 38360072 10.1016/j.bbadis.2024.167056

[14] Y. Huang , T. Chen , M. Jiang , et al., “E3 Ligase TRIM65 Alleviates Intestinal Ischemia/Reperfusion Injury Through Inhibition of TOX4‐Mediated Apoptosis,” Cell Death & Disease 15, no. 1 (2024): 29. 38212319 10.1038/s41419-023-06410-x PMC10784301

[15] S. Wei , X. Huang , Q. Zhu , et al., “TRIM65 Deficiency Alleviates Renal Fibrosis Through NUDT21‐Mediated Alternative Polyadenylation,” Cell Death and Differentiation 31, no. 11 (2024): 1422–1438. 38951701 10.1038/s41418-024-01336-z PMC11519343

[16] X. Li , J. Pan , H. Li , et al., “DsbA‐L Interacts With VDAC1 in Mitochondrion‐Mediated Tubular Cell Apoptosis and Contributes to the Progression of Acute Kidney Disease,” EBioMedicine 76 (2022): 103859. 35124430 10.1016/j.ebiom.2022.103859 PMC8829058

[17] G. Nowak , J. Megyesi , and W. J. Craigen , “Deletion of VDAC1 Hinders Recovery of Mitochondrial and Renal Functions After Acute Kidney Injury,” Biomolecules 10, no. 4 (2020): 585. 32290153 10.3390/biom10040585 PMC7226369

[18] M. E. French , C. F. Koehler , and T. Hunter , “Emerging Functions of Branched Ubiquitin Chains,” Cell Discovery 7, no. 1 (2021): 6. 33495455 10.1038/s41421-020-00237-y PMC7835216

[19] K. N. Swatek and D. Komander , “Ubiquitin Modifications,” Cell Research 26, no. 4 (2016): 399–422. 27012465 10.1038/cr.2016.39 PMC4822133

[20] Y. Xiao , R. Liu , N. Li , Y. Li , and X. Huang , “Role of the Ubiquitin‐Proteasome System on Macrophages in the Tumor Microenvironment,” Journal of Cellular Physiology 239, no. 2 (2024): e31180. 38219045 10.1002/jcp.31180

[21] L. D. Zorova , V. A. Popkov , E. Y. Plotnikov , et al., “Mitochondrial Membrane Potential,” Analytical Biochemistry 552 (2018): 50–59. 28711444 10.1016/j.ab.2017.07.009 PMC5792320

[22] W. Y. Ding , V. Kuzmuk , S. Hunter , et al., “Adeno‐Associated Virus Gene Therapy Prevents Progression of Kidney Disease in Genetic Models of Nephrotic Syndrome,” Science Translational Medicine 15, no. 708 (2023): eabc8226. 37556557 10.1126/scitranslmed.abc8226

[23] A. D. Cowan and A. Ciulli , “Driving E3 Ligase Substrate Specificity for Targeted Protein Degradation: Lessons From Nature and the Laboratory,” Annual Review of Biochemistry 91 (2022): 295–319. 10.1146/annurev-biochem-032620-104421 35320687

[24] L. Yang and H. Xia , “TRIM Proteins in Inflammation: From Expression to Emerging Regulatory Mechanisms,” Inflammation 44, no. 3 (2021): 811–820. 33415537 10.1007/s10753-020-01394-8

[25] P. Duann , H. Li , P. Lin , et al., “MG53‐Mediated Cell Membrane Repair Protects Against Acute Kidney Injury,” Science Translational Medicine 7, no. 279 (2015): 279ra236. 10.1126/scitranslmed.3010755 PMC4524523 25787762

[26] X. Sun , N. Huang , P. Li , et al., “TRIM21 Ubiquitylates GPX4 and Promotes Ferroptosis to Aggravate Ischemia/Reperfusion‐Induced Acute Kidney Injury,” Life Sciences 321 (2023): 121608. 36958437 10.1016/j.lfs.2023.121608 PMC11483487

[27] X. K. Li , X. Z. Xu , Q. Cong , et al., “Tri‐Domain Proteins 27 Reduce Inflammation and Apoptosis in HK‐2 Cells and Protect Against Acute Kidney Injury in Mice,” European Review for Medical and Pharmacological Sciences 24, no. 23 (2020): 12258–12266. 33336745 10.26355/eurrev_202012_24018

[28] Y. F. Yang , M. F. Zhang , Q. H. Tian , and C. Z. Zhang , “TRIM65 Triggers Beta‐Catenin Signaling via Ubiquitylation of Axin1 to Promote Hepatocellular Carcinoma,” Journal of Cell Science 130, no. 18 (2017): 3108–3115. 28754688 10.1242/jcs.206623

[29] Y. Wang and Q. Zhang , “Long Noncoding RNA MALAT1 Knockdown Inhibits Proliferation, Migration, and Invasion and Promotes Apoptosis in Non‐Small‐Cell Lung Cancer Cells Through Regulating miR‐515‐3p/TRIM65 Axis,” Cancer Biotherapy & Radiopharmaceuticals (2020). 10.1089/cbr.2020.3730 33395541

[30] X. Zeng , X. Deng , Y. Ni , et al., “LPS Inhibits TRIM65 Expression in Macrophages and C57BL/6J Mouse by Activating the ERK1/2 Signaling Pathway,” Experimental and Therapeutic Medicine 25, no. 4 (2023): 188. 37021067 10.3892/etm.2023.11887 PMC10068263

[31] Q. Zhang , Y. Li , Q. Zhu , et al., “TRIM65 Promotes Renal Cell Carcinoma Through Ubiquitination and Degradation of BTG3,” Cell Death & Disease 15, no. 5 (2024): 355. 38777825 10.1038/s41419-024-06741-3 PMC11111765

[32] C. Tang , J. Cai , X. M. Yin , J. M. Weinberg , M. A. Venkatachalam , and Z. Dong , “Mitochondrial Quality Control in Kidney Injury and Repair,” Nature Reviews Nephrology 17, no. 5 (2021): 299–318. 33235391 10.1038/s41581-020-00369-0 PMC8958893

[33] H. H. Szeto , “Pharmacologic Approaches to Improve Mitochondrial Function in AKI and CKD,” Journal of the American Society of Nephrology 28, no. 10 (2017): 2856–2865. 28778860 10.1681/ASN.2017030247 PMC5619975

[34] H. Hu , L. Guo , J. Overholser , and X. Wang , “Mitochondrial VDAC1: A Potential Therapeutic Target of Inflammation‐Related Diseases and Clinical Opportunities,” Cells 11, no. 19 (2022): 3174. 36231136 10.3390/cells11193174 PMC9562648

[35] A. Magri , S. Reina , and V. De Pinto , “VDAC1 as Pharmacological Target in Cancer and Neurodegeneration: Focus on Its Role in Apoptosis,” Frontiers in Chemistry 6 (2018): 108. 29682501 10.3389/fchem.2018.00108 PMC5897536

[36] Y. T. Wang , T. Y. Liu , C. H. Shen , et al., “K48/K63‐Linked Polyubiquitination of ATG9A by TRAF6 E3 Ligase Regulates Oxidative Stress‐Induced Autophagy,” Cell Reports 38, no. 8 (2022): 110354. 35196483 10.1016/j.celrep.2022.110354

[37] X. Chen , Q. Zhao , Y. Xu , et al., “E3 Ubiquitin Ligase MID1 Ubiquitinates and Degrades Type‐I Interferon Receptor 2,” Immunology 167, no. 3 (2022): 398–412. 35794827 10.1111/imm.13544

[38] S. Geisler , K. M. Holmstrom , D. Skujat , et al., “PINK1/Parkin‐Mediated Mitophagy Is Dependent on VDAC1 and p62/SQSTM1,” Nature Cell Biology 12, no. 2 (2010): 119–131. 20098416 10.1038/ncb2012

[39] S. J. Ham , D. Lee , H. Yoo , K. Jun , H. Shin , and J. Chung , “Decision Between Mitophagy and Apoptosis by Parkin via VDAC1 Ubiquitination,” PNAS 117, no. 8 (2020): 4281–4291. 32047033 10.1073/pnas.1909814117 PMC7049170

[40] N. N. Wu , L. Wang , L. Wang , et al., “Site‐Specific Ubiquitination of VDAC1 Restricts Its Oligomerization and Mitochondrial DNA Release in Liver Fibrosis,” Experimental & Molecular Medicine 55, no. 1 (2023): 269–280. 36658227 10.1038/s12276-022-00923-9 PMC9898252

[41] Y. Dong , Q. Zhang , J. Wen , et al., “Ischemic Duration and Frequency Determines AKI‐to‐CKD Progression Monitored by Dynamic Changes of Tubular Biomarkers in IRI Mice,” Frontiers in Physiology 10 (2019): 153. 30873045 10.3389/fphys.2019.00153 PMC6401609

