# Supplementary material for: Tripartite Motif Containing 65 Deficiency Confers Protection Against Acute Kidney Injury via Alleviating Voltage‐Dependent Anion Channel 1–Mediated Mitochondrial Dysfunction
Source: MedComm (2020). 2025 Apr 22;6(5):e70149. doi: 10.1002/mco2.70149 (PMC12013732; doi:10.1002/mco2.70149)
Supplement: Supplementary file 1 — Supporting Information [file MCO2-6-e70149-s001.pdf]

Tripartite motif containing 65 deficiency confers protection against acute kidney injury via alleviating voltage-dependent anion channel 1 mediated mitochondrial dysfunction

Tao Chen[1,2,3†], Yang Zhang[1†], Liting Ding[2], Chenlu Xiong[1], Chao Mei[1], Sisi Wei[1], Ming Jiang[1,2], Yingjie Huang[1,2], Jianrong Chen[4], Tao Xie[2], Qing Zhu[2], Qi Zhang[2], Xuan Huang[2*], Shibiao Chen[1*], Yong Li[1*]

[1]Department of Anesthesiology, the First Affiliated Hospital, Jiangxi Medical College, Nanchang University, Nanchang 330006, China;

[2]The National Engineering Research Center for Bioengineering Drugs and the Technologies, Jiangxi Provincial Key Laboratory of Bioengineering Drugs, Institute of Translational Medicine, Jiangxi Medical College, Nanchang University, Nanchang 330031, PR China;

[3]Department of Anesthesiology, Sir Run Run Shaw Hospital, School of Medicine, Zhejiang University, Hangzhou, 310016, China;

[4]Department of Endocrinology, The First Affiliated Hospital, Jiangxi Medical College, Nanchang University, Nanchang 330006, China;

[†]Contributed equally

***Correspondence:**

Address correspondence to: Yong Li, The First Affiliated Hospital, Jiangxi Medical College, Nanchang University, 17 Yongwaizheng Street, Donghu District, Nanchang 330006, China; Phone: +86-13979131536; Electronic address: liyong@ncu.edu.cn;
Address correspondence to: Shibiao Chen, The First Affiliated Hospital, Jiangxi Medical College, Nanchang University, 17 Yongwaizheng Street, Donghu District, Nanchang 330006, China; Phone: +86-13870982918; Electronic address: ndyfy00763@ncu.edu.cn;

Address correspondence to: Xuan Huang, Institute of Translational Medicine, Jiangxi Medical College, Nanchang University, 1299 Xuefu Road, Honggutan District, Nanchang 330031, China; Phone: +86-13807058627; Electronic address: huangxuan@ncu.edu.cn.

Materials and Methods

Cisplatin-induced AKI model

Following a 12-hour fast, female mice aged 6-8 weeks were weighed and anesthetized with isoflurane (2%). The model group and control group were intraperitoneally injected with 20 mg/kg cisplatin (MCE, USA) and an equal amount of PBS (pH 7.4), respectively. All mice were anesthetized and euthanized three days later. Biochemical and pathological examinations were conducted on the mice through ocular blood sampling and nephrectomy, respectively.

Mouse Adeno-associated virus (AAV) injection

AAV represents a significant gene delivery vector, and numerous injection methods have been developed in recent years. In this study, we referenced the research methods of Sisi Wei, *et al.*[1] for intra-renal pelvis injection and procured AAV from OBiO Technology (Shanghai, China). The AAV control vector (pAAV-CMV-MCS-EF1-GdGreen WPRE) and the recombinant AAV-VDAC1 (pAAV-CMV-VDAC1-EF1-GdGreen WPRE), which was used to overexpress VDAC1, were injected into the intra-renal pelvis. AAV was diluted in a saline solvent at a concentration of 50 μL per 10^{11} vector genomes (v.g.) per mouse. Following a 21-day period, all mice were subjected to the I/R AKI model.

Detection of kidney function

Whole blood samples were obtained from the eyeballs of anesthetized mice, and the serum was collected by centrifugation after clotting at room temperature. Serum creatinine (Cr) and blood urea nitrogen (BUN) were measured using an automated biochemistry analyzer (Chemray 800, Shenzhen, China), which serves as an indicator of renal function.

Glomerular filtration rate (GFR) test

Before the commencement of the experiment, all mice were shaved and depilation cream was applied. On the subsequent day, the fluorescently labelled exogenous tracer

FITC-sinistrin was administered via the tail vein. It is crucial to emphasize that a single injection is essential to minimize experimental errors. The mini device (MediBeacon, Germany) was attached to the skin of the back and not the spine/limbs, and the fluorescence intensity changes of the subcutaneous tissue were continuously monitored in real-time. Once the injection was complete, the mice were released and the device was detached. After the experiment, a professional software was utilized to the data readout.

Lentivirus Infection, Calcium Phosphate, and Liposomal Transfection

The HEK293T cells were achieved approximately 70% confluence and treated with 10 mM chloroquine (CQ) (Sigma Aldrich, USA) for one hour prior to transfection. The mixture comprising plasmids, $CaCl_2$, and 2×HBS was agitated twice to produce bubbles and incubated on ice for 20 minutes. Thereafter, it was dropped into the medium. The harvesting of protein or supernatant can be conducted 48 to 72 hours post-transfection. The lentivirus overexpressing TRIM65 and the control lentivirus were obtained by the calcium phosphate transfection technique, as previously described[2]. In brief, pLVX-IRES-ZsGFP or pLVX-IRES-TRIM65-ZsGFP were co-transfected with packaging plasmids pMD2G and pSPAX2 into HEK293T cells, and the viral supernatant was collected 72 hours later. Subsequently, the lentivirus was filtered using a 0.45 μm Millipore Express® PES Membrane Filtration Unit (Millipore, USA), after which it was stored at -80°C. HK-2 cells were inoculated in 6-well plates until they reached 50% confluence on the second day. The mixture of lentivirus, complete medium, polybrene, and HEPES was added and incubated overnight. The lentivirus of si-TRIM65 and the negative control were purchased from OBiO Technology (Shanghai, China). Gene expression was then detected by fluorescence microscopy (Invitrogen, USA) and immunoblotting after three days.

HK-2 cells were inoculated into 12-well plates and allowed to reach 80% confluence the day preceding transfection. The Flag-tagged TRIM65 plasmid and Hieff Trans® Liposomal Transfection Reagent (YEASEN, Shanghai, China) were diluted separately with OPTI-MEM (Gibco, USA). Following a five-minute incubation period, the two

solutions were gently mixed and incubated for a further twenty minutes to form the DNA-liposome complexes. These complexes were then added to the cell culture plate. After four hours, the cells were washed with fresh complete medium and the expression of the gene was analyzed twenty-four hours later.

Yeast two-hybrid system

The yeast two-hybrid screening was conducted in accordance with the previously established protocol[3]. The cDNA of TRIM65 was cloned into the pGBKT7 vector as a bait, and was subsequently transformed into the Y2HGold strain using the Yeastmaker™ Yeast Transformation System 2. Subsequently, the Y2HGold strain containing pGBKT7-TRIM65 was mated with the yeast strain Y187, which had been transformed with the Universal Human Mate & Plate™ Library (Clontech Laboratories). Blue colonies that grew on a high selectivity medium (SD/-Ade/-His/-Leu/-Trp/X-α-Gal, QDO/X/A) were considered positive. Positive cDNA candidates were then subjected to PCR followed by first-generation sequencing to identify target genes.

RT-qPCR

Total RNA was extracted from kidney tissues in mice using a TRIzol reagent (T9108, Takara) in accordance with the manufacturer's instructions. cDNA was then reverse-transcribed from 1 μg RNA using Hifair® V one-step RT-gDNA digestion SuperMix for qPCR (YEASEN, Shanghai, China). Subsequently, quantitative PCR (qPCR) analysis of target genes was conducted and analyzed using Hieff® qPCR SYBR Green Master Mix (No Rox) (YEASEN, Shanghai, China) and CFX96 Connect system (Bio-Rad, CA, USA). Primers were designed using Primer Premier 5 and are listed in Supplementary Table 1. The results were normalized to β-actin and quantified by the $2^{-\Delta\Delta Ct}$ method.

Western blot

All tissues and cells were homogenized in a mild lysis buffer (0.5% lubrol-px, 50 mM KCl, 2 mM CaCl2, 20% glycerol, 50 mM Tris-HCl, phosphatase inhibitors 3 mM

sodium vanadate and 2 mM NaF) containing protease inhibitor cocktail (GLPBIO, USA, GK10014, 1:1000) and a nonionic detergent lubrol-px (Sigma-Aldrich, USA, SLBD6973V), before being subjected to centrifugation at 12,000 g at 4 °C for 15 minutes. The protein concentration was determined using the PieceTM BCA Protein Assay Kit (Thermo Scientific, USA). Following the addition of 2× loading buffer (comprising 2.1% SDS, 0.01% bromophenol blue, 26.3% glycerol, 6.58% Tri-HCl pH 6.8 and 0.5% β-mercaptoethanol), whole cell lysates (WCL) were collected and loaded onto sodium dodecyl sulfate polyacrylamide (SDS-PAGE), which were then transferred to nitrocellulose (NC) membrane (PALL Corporation, USA). Subsequently, the membrane was sealed with 5% non-fat milk at room temperature for one hour. The corresponding primary antibodies were incubated by the following protocol: TRIM65 (Atlas Antibodies, Sweden; HPA021578, 1:1000), VDAC1 (Proteintech, China; 55259-1-AP, 1:1000), KIM-1 (R&D systems, USA; AF1817, 1:1000), NGAL (R&D systems, USA; AF1857, 1:1000), GAPDH (Proteintech, China; 60004-1-Ig, 1:10,000), β-actin (Proteintech, China; 66009-1-Ig, 1:10,000), α-tubulin (Proteintech, China; 66031-1-Ig, 1:10,000), Flag (Proteintech, China; 66008-4-Ig, 1:5000), HA (Proteintech, China; 66006-2-Ig, 1:10,000), GFP (Proteintech, China; 66002-1-Ig, 1:10,000). On the subsequent day, the membranes were rinsed three times with PBS-T buffer for ten minutes and subsequently incubated with the corresponding horseradish peroxidase (HRP)-bound secondary antibodies (Thermo Scientific, USA; 31460, 1:2000 and 31430, 1:2000) at room temperature for one and a half hours. Finally, the bands were visualized using an enhanced ECL chemiluminescent substrate kit (YEASEN, Shanghai, China) under an ECL detection system (TANON 5500, China). The protein quantification was analyzed with ImageJ.

Co-IP and ubiquitination assay

The HEK293T cells were cultured until they reached approximately 70% confluence and then transfected with different plasmids using the standard calcium phosphate technique. Following a 48-hour incubation period, the cells were harvested using lysis buffer, which was applied for a period of four hours in the presence of 10 μM MG132

(Sigma Aldrich, USA), a proteasome inhibitor. The specific 1 μg tag antibodies were employed for the immunoprecipitation of cell extract protein, resulting in the formation of immune complexes. Subsequently, the complexes were added to 100 μL of pre-activated Protein A/G Agarose (Beyotime, China) at 4°C overnight. Subsequently, the beads were rinsed four times with lysis buffer and added to the loading buffer for western blot detection with the corresponding antibodies. In the ubiquitination assay, Flag-TRIM65 or its RING domain mutant, HA-Ub or its mutants (HA-Ub-K6O, HA-Ub-K11O, HA-Ub-K27O, HA-Ub-K29O, HA-Ub-K33O, HA-Ub-K48O, HA-Ub-K63O, HA-Ub-K48R, and HA-Ub-K63R), along with the GFP-VDAC1 construct and its K to R mutants, were transfected into HEK293T cells. The resulting lysates were subjected to immunoprecipitation using the indicated tag antibodies, which were then bound to Protein A/G Agarose Beads. The prospective protein was subsequently analyzed by immunoblotting.

GST pull-down assay

To elucidate the physical interaction between TRIM65 and VDAC1, Glutathione Sepharose^{TM} 4B (GE Healthcare, Sweden) was initially washed twice with pre-cooled PBS containing 1% cocktail to be activated. The GST and GST-TRIM65 purified in E. coli BL21(DE3) with isopropyl thio-β-d-galactoside (IPTG) induction at 16 °C were added to Glutathione Sepharose^{TM} 4B slowly and steadily, with the utmost care taken to avoid the formation of bubbles, and mixed for 4 hours. Following a wash with PBS and centrifugation, lysates of HEK293T cells transfected with HA-VDAC1 were combined with GST or GST-TRIM65 and incubated overnight at 4 °C. The complexes were then centrifuged and rinsed thoroughly prior to immunoblotting with the appropriate antibodies.

Immunofluorescence

Approximately 20% of confluence HK-2 cells were inoculated onto the coverslips. The cells were fixed in 4% paraformaldehyde for 30 minutes, permeabilized with 0.25% Triton X-100 (Servicebio, China) for 5 minutes, and blocked with 1% bovine serum

albumin (BSA) for 1 hour at room temperature. Subsequently, the cells were incubated with anti-TRIM65 (Atlas Antibodies, Sweden; HPA021578, 1:100) and anti-VDAC1 (Proteintech, China; 66345-1-Ig, 1:100) antibodies for 16-18 hours at 4 °C. The following fluorescent secondary antibodies were employed: The CoraLite594 conjugated goat anti-mouse IgG H&L (1:400) and CoraLite488-conjugated goat anti-rabbit IgG (H+L) antibody (1:400) were incubated at room temperature in a humid and dark environment for 2 hours. The nuclei were stained with 4',6-diamidino-2-phenylindole (DAPI; BOSTER, China) and the cells were fixed with mounting medium (BOSTER, China). The images were analyzed using high-resolution laser confocal microscopy (Leica, Germany).

References:

1. Wei S, Huang X, Zhu Q, Chen T, Zhang Y, Tian J*, et al.* TRIM65 deficiency alleviates renal fibrosis through NUDT21-mediated alternative polyadenylation. *Cell Death Differ* 2024, **31**(11)**:** 1422-1438.

2. Huang Y, Chen T, Jiang M, Xiong C, Mei C, Nie J*, et al.* E3 ligase TRIM65 alleviates intestinal ischemia/reperfusion injury through inhibition of TOX4-mediated apoptosis. *Cell Death Dis* 2024, **15**(1)**:** 29.

3. Zhang Q, Li Y, Zhu Q, Xie T, Xiao Y, Zhang F*, et al.* TRIM65 promotes renal cell carcinoma through ubiquitination and degradation of BTG3. *Cell Death Dis* 2024, **15**(5)**:** 355.

Supplementary Figures

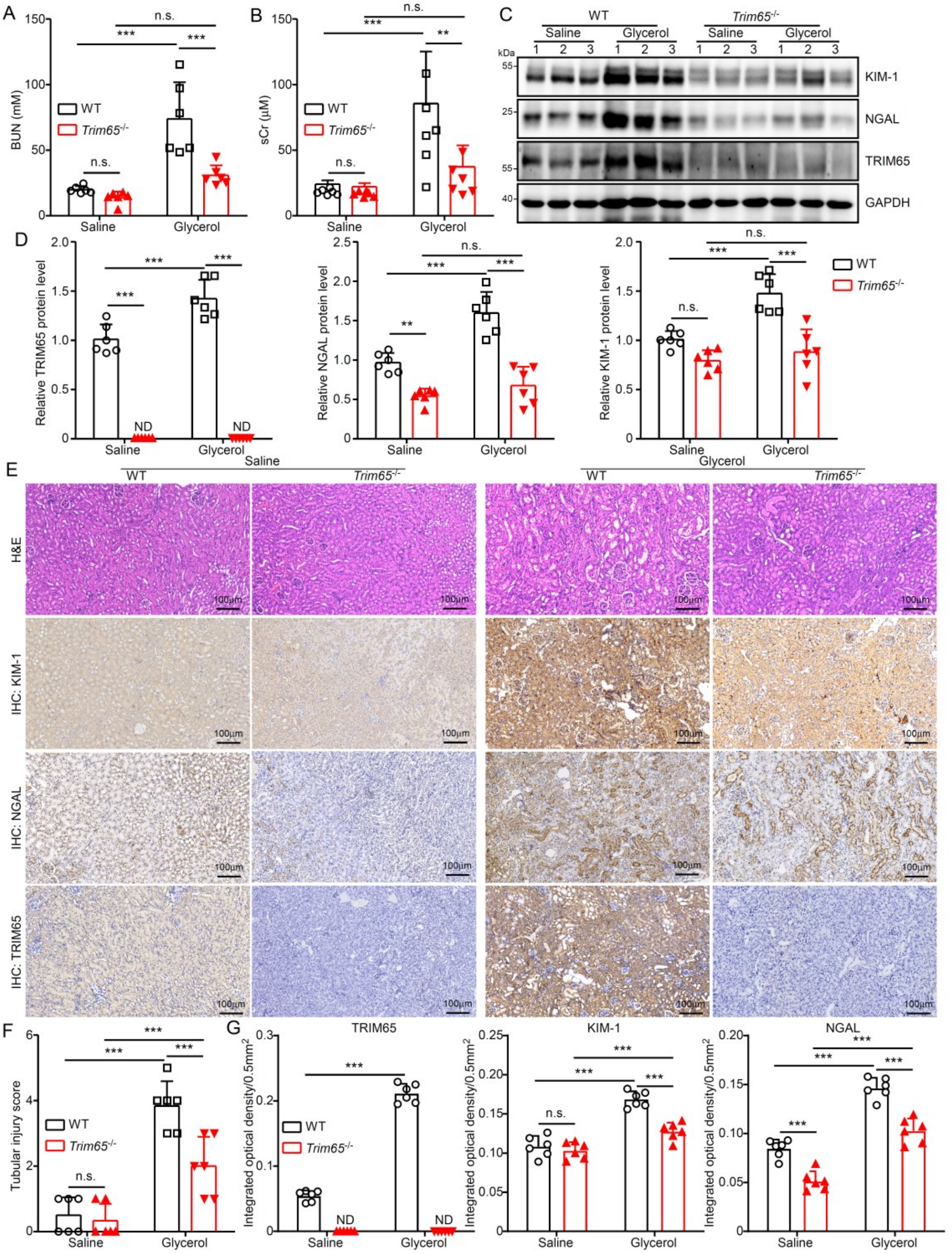

Figure S1. ***Trim65* gene deletion protected against rhabdomyolysis-induced AKI**

(A) The impact of *Trim65* gene deletion on BUN levels in mice treated intramuscularly with 50% glycerol or an equal volume of saline for 24 hours. (B) The impact of *Trim65* gene deletion on sCr levels in the peripheral blood of mice treated intramuscularly with 50% glycerol or an equal volume of saline for 24 hours. (C) Western blot analysis of kidney tissue extracts obtained from mice following a 24-hours treatment period with either normal saline or a 50% glycerol intramuscular injection. (D) Using the ImageJ software to conduct a grayscale analysis of the western blot bands of the KIM-1, TRIM65, and NGAL, with GAPDH as the internal reference. (E) Representative

H&E staining of kidney sections and IHC staining detection of TRIM65, KIM-1, and NGAL in four groups of mice (n=6) are presented. Following intramuscular administration of 8 mL/kg 50% glycerol or an equivalent volume of saline to both hind limbs, one side of the kidney tissue was harvested and fixed with 4% paraformaldehyde 24 hours following the glycerol challenge. The scale bar represents a length of 100 μm. (F) The renal tubular injury score. (G) The degree of staining and the positive range of TRIM65, KIM-1, and NGAL are evaluated under an optical microscope, and the IHC is scored accordingly. The study included six mice per group. All data were subjected to the two-way ANOVA, and Tukey's post hoc test was used for multiple group comparisons. Statistical significance was indicated by ** $p < 0.01$, *** $p < 0.001$, n.s. indicates not significance.

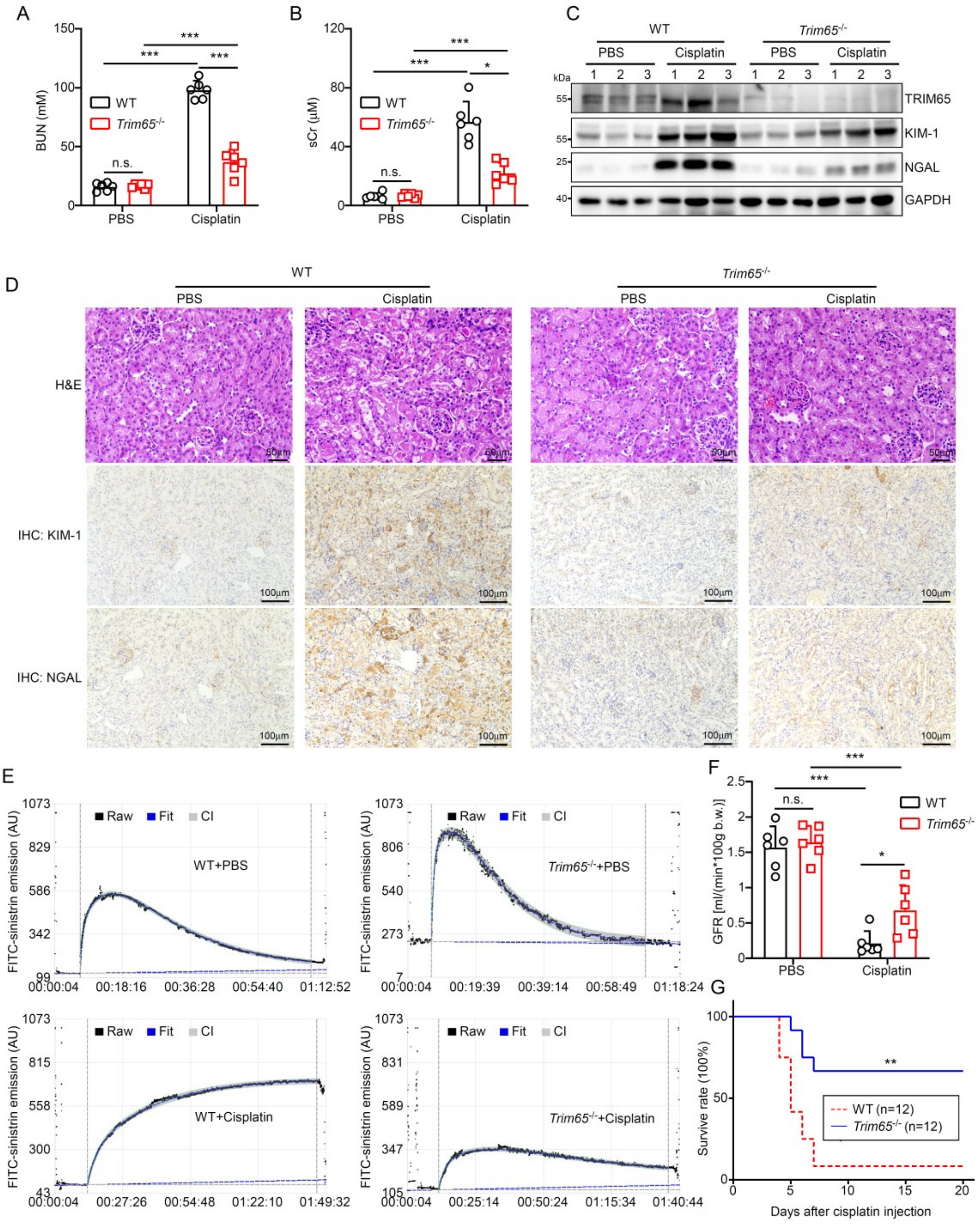

Figure S2. Genetic deletion of TRIM65 alleviated cisplatin-induced AKI

(A) The changes in BUN in mice treated with 20 mg/kg cisplatin or an equivalent volume of phosphate-buffered saline for 72 hours were evaluated. (B) The changes in sCr in mice following intraperitoneal administration of 20 mg/kg cisplatin or an equivalent volume of phosphate-buffered saline for 72 hours were examined. (C) Western blot analysis was employed to assess the expression levels of TRIM65, KIM-1, and NGAL proteins in injured renal tissues. (D) Representative H&E staining of kidney sections, as well as IHC staining for the detection of KIM-1 and NGAL, were conducted. Tissue samples were obtained 72 hours after intraperitoneal administration of 20 mg/kg cisplatin or an equal volume of phosphate-buffered saline. Scale bar: 100 μm. (E) Glomerular

filtration rate (GFR) measurement. (F) Quantification of glomerular filtration rate measurement. (G) The survival rate of the WT and *Trim65*$^{-/-}$ mice after intraperitoneal injection of 20 mg/kg cisplatin (n=12). All data were subjected to two-way ANOVA and Tukey's post hoc test was employed for multiple group comparisons. Statistical significance was indicated by ** $p < 0.01$, *** $p < 0.001$, n.s. indicates not significance.

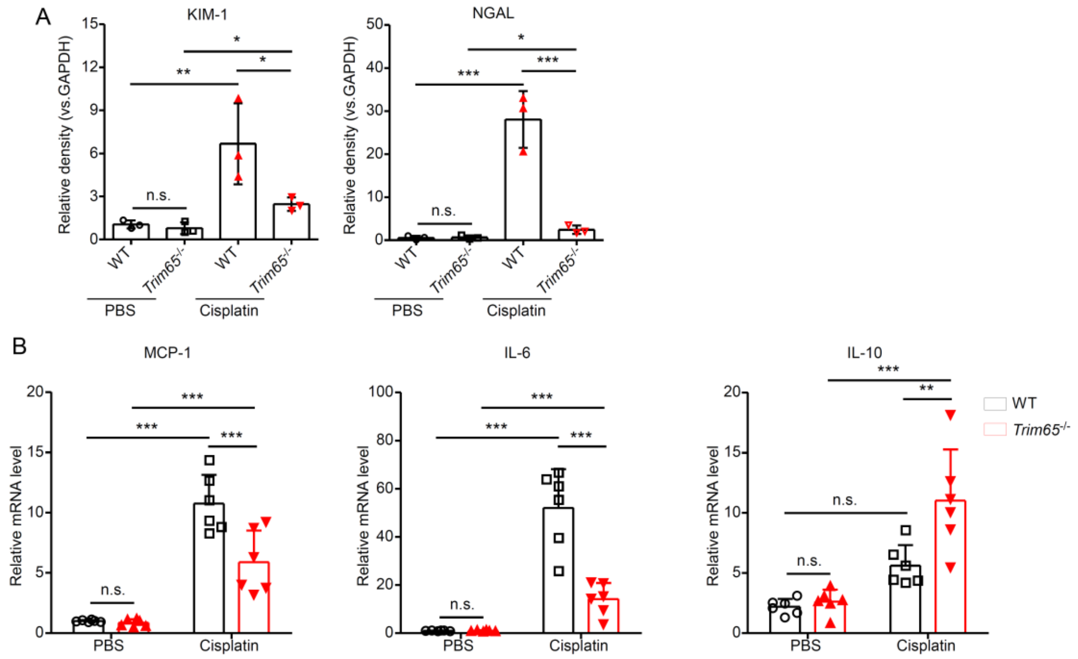

Figure S3. Genetic deletion of TRIM65 alleviated cisplatin-induced renal damage and inflammation

(A) The KIM-1 and NGAL western blot band in Figure S1C was quantified using ImageJ software. (B) The expression of inflammatory factors such as MCP-1, IL-6 and IL-10 in the renal tissues of mice in each group was detected by qRT-PCR. Statistical significance was indicated by * $p < 0.05$, ** $p < 0.01$, *** $p < 0.001$, n.s. indicates not significance.

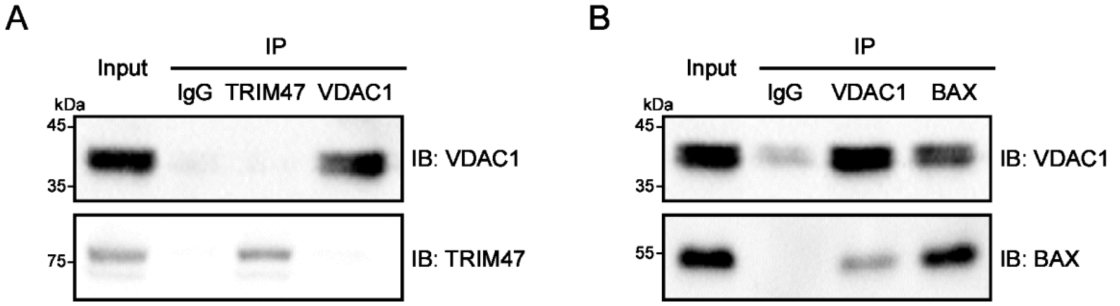

Figure S4. Bax, but not TRIM47, was observed to bind VDAC1 in HK-2 cells.

(A) The interaction between TRIM47 and VDAC1 was examined by co-immunoprecipitation (Co-IP) and western blot analysis in HK-2 cells, with IgG serving as a negative control. (B) The interaction between BAX and VDAC1 was determined by Co-IP and western blot analysis in HK-2 cells, with IgG serving as a negative control.

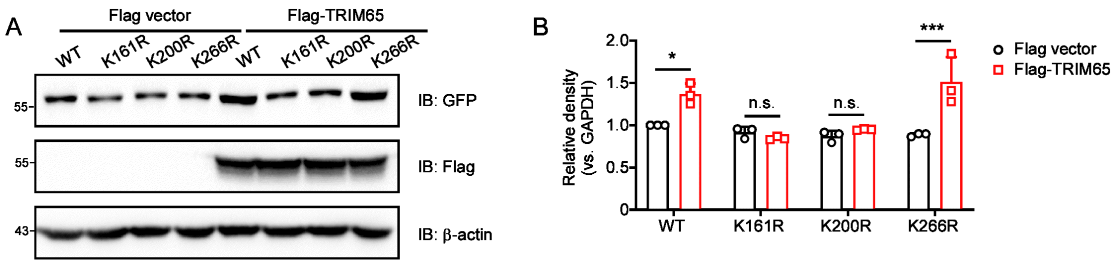

Figure S5. VDAC1 stability is dependent on TRIM65-mediated ubiquitination modifications

(A) The impact of TRIM65 protein overexpression on the WT and mutants of VDAC1 protein level. HEK293T cells were co-transfected with GFP-VDAC1 (WT) and its mutant (K161R, K200R, and K266R) and Flag-TRIM65. Following a 48-hour incubation period, cell lysates were subjected to immunoblot analysis using Flag and GFP antibodies. β-actin was employed as a standardization control. (B) The protein levels of GFP-VDAC1 and its mutants were quantified using ImageJ software. Statistical significance was indicated by * $p < 0.05$, *** $p < 0.001$, n.s. indicates not significance.

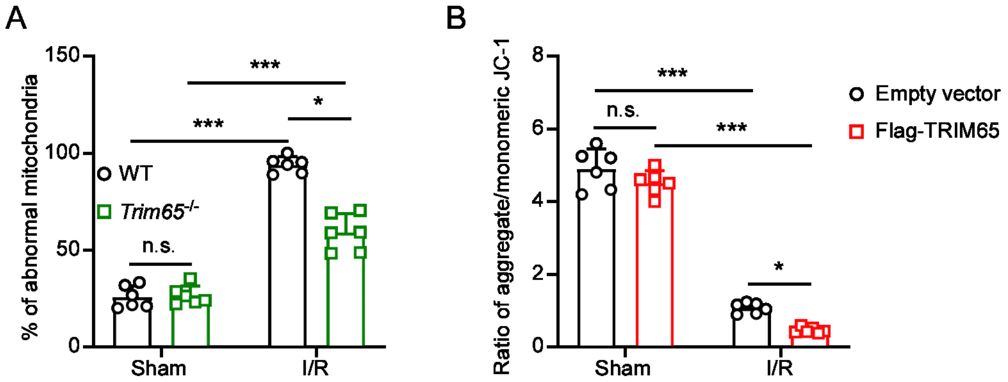

Figure S6. TRIM65 aggravated mitochondrial dysfunction

(A) Percentage of mitochondria exhibiting abnormal morphology in the kidney tissues of mice that had undergone either renal I/R or sham surgery for 24 hours. (B) The ratio of aggregate (red)/monomeric (green) JC-1 in HK-2 cells expressing Lenti-control or Lenti-TRIM65 under H/R injury or normoxia was observed. Statistical significance was indicated by * $p < 0.05$, *** $p < 0.001$, n.s. indicates not significance.

Supplementary Table 1

The primers used in this study

Primer name	Forward primer (5' - 3')	Reverse primer (3' - 5')
mTRIM1	CCGGGAACAATATGGGTGAGA	AGGCTGTGAGCACAAGGAAG
mTRIM2	TGGGCTCATTTGTCTGAGGG	CTGCACGCTGCTGGGTT
mTRIM3	CCATGAAGGCAAGACAATGGA	TGTGCTGCTCCACCACATC
mTRIM5	AAAACCATCGCCAGGGAACA	AGGGTCACCTGAACCCAGTA
mTRIM6	CTCCTTGGCTGCAAGTCCTC	AGCTTGCTTAGAAAAAGAATCCAAT
mTRIM7	GACTCCAGCGGTGCCTG	GAATCCAGCAACTCCTTGGC
mTRIM8	GCTGCCCGCAGCACAAC	CTGCTTCATGAGCATCTTCCGTATC
mTRIM9	CAACCACCCTGATCCAGCAT	TCCCTCCTTCAGTTATTGCCC
mTRIM10	CGGTTCAGCACCCTGATTGA	AGAGTGCTTCTGATATCCGTCA
mTRIM11	TGGCAGAAATGGCACGACG	CTTCTCCAGCCTCCCCTTGAG
mTRIM15	CCTGAGCGAGACCTACTGTG	ACTCCTGAGACGATCCCTGT
mTRIM16	CGGTGTATGCAGTTGGACCT	CCTCTGACACCGACACCAAA
mTRIM17	GCAAGGCTCCTTCCTTGAGC	CACAGGCAGTCATCACAGGA
mTRIM18	AAGAGCAGTCTGTACTTGCCC	CTGATCAGCTATCGGCTTCC
mTRIM19	ATAGCAGCAGTGAGTCCAGC	GCTGGCTAATTTTCTGGGTTTC
mTRIM20	TGGAAACCTTCATTCATTGGGA	CACGGTAACTGCAGGAAGAAG
mTRIM21	AGGCTCCTGTCATTCAGATTCCA	GAACTGCCCCCATTCTTCCC
mTRIM23	AGCACCAAGGTCACAAGCATT	TGAAGGTCCGTATGCAGTGAG
mTRIM24	ATCTCTCATCAGCATCCGCC	TTGCAAGGGGTTGGGCTTTA
mTRIM25	GGAGACCAGACACAGTCCAC	CGGGAGCAACAGGGGTTT
mTRIM26	CTGCACTACACAGGACACCA	GTGAGGGATGGCTTTCCCAA
mTRIM27	GGAGGGCTTCAAGGAGCAA	GCTGCTCAAACTCCCAGACA
mTRIM28	TGCTGCCCTGTCTACATTCG	ACTGGACAATCCACCATAGCG
mTRIM29	AAGGCTTTCCCTCCCTCCT	CCGGTAGTGAGACAGCAGAG
mTRIM32	GGGCGGTCAGCAGGAATC	GCATCCAGGTTCAGGTGAGA
mTRIM33	ACACAAGAGATTAATTTGAAGG GTG	TGGCAAAGGAGTGAAGGTCC
mTRIM34	GGAGGTGAGGGTCCAAAGATTC	GCTGGATGGCTAGGCAGATT
mTRIM35	CTAGCAGAACAGACCGAGGC	GGCTCTTGTGTTTCATGAGGA
mTRIM37	GCCCCTGAGGAAGGAATGAG	ACTGGAATGTGAATCTTCATCGG

Primer name	Forward primer (5’ - 3’)	Reverse primer (3’ - 5’)
mTRIM38	AGGCTGATCCCCTGAAGGTT	CAGGATCACGCTGACTTGGTG
mTRIM39	TGGAGGGCAAGTGCTTACAG	TCCAGAGTCACATCCGCAAT
mTRIM40	GAAGAACCGGCTCATCTGCT	GAACCTTGTCAGTGACCCTCA
mTRIM41	GGCTGCAAGAGTGGACCTG	CCCACCTCTACCTCCCAGTAG
mTRIM43	CTCAACTCCTTCCGAGTGAAGA	CCTTCAAATCCACCTCCCAGT
mTRIM44	GGGCTGGATTTGAGCACCTA	CACCGGAATCTTTGCTTCTCAG
mTRIM45	CCAGGCTCATAGGCGACAG	CTACACAGTCCCGACACACC
mTRIM46	GAAGAGAGGGACCAAGACAAGC	CCCACCATGGCCTATGTACC
mTRIM47	GGCTACAGAAACTCGGCTCA	CACGATGTAGGCAAACTTGAGG
mTRIM50	CTACAGCCGCATGAAGGAAG	TGTTGTTCACCAGTTTGCCG
mTRIM54	GTGCCAGACCATTGAGGACA	CTTGAAGCAGTTCGCCCTTG
mTRIM56	AGGGCATTTTCCCAACTAGCA	TTGCCAGCCAACACTACTCT
mTRIM58	AGTGGGACTGATGAGTGGGT	AATGAAGCCTCGGGCAGTAG
mTRIM63	ATGAGTGAGACACGCTCTGG	TGGGGAGCCCTATGCTAGTC
mTRIM65	GCTCTGGCAGAATTATCGCAATC	AAATGGTGTGTCACCCGCTG
mTRIM66	ATGAGTGAGACACGCTCTGG	TGGGGAGCCCTATGCTAGTC
mTRIM67	TGGAGCCCAAAGTATCTGCG	GGGGTACAGGTGGCAATTTCA
mTRIM68	TACTCCCGCCTTGTCGTTTC	GATGCATTGGCTGCCCAAAA
mTRIM71	GACCGCATTATGTTCACGCC	TGACAGTGAAAGAGGCCACC
mTRIM72	AGGTAGTTACAGGATGGGGCT	CATGGTGAGCCTGGGAAGAG
mTRIM75	CTGATGGCACATGTCGAGGT	ACAGAAGTTGTGTCCGCACT
mTRIM80	TCGCCAACAGGAGTGATGAG	TTGGTGCCTAGCAAGGGTTC
hTRIM65	GAGAGCCAGCCTGGAGGTTA	CCGAGTTCTGGATCTGGCTG
mTRIM65	CTGGAACTCCCATCTGCTCTGCT	GGGACGAGTGTGGACAGGACAGTT
mTRIM65-Wt/He	CAGGAGATTCAGTAGCCTGCTTCAGG	
EGFP-VDAC1-Xhol	GCGCCTCGAGCTATGGCTGTGCCACCCAC	
EGFP-VDAC1-		GCGCGGATCCTTATGCTTGAAATTCCAGTCCTAG

Primer name	Forward primer (5’ - 3’)	Reverse primer (3’ - 5’)
BamHl VDAC1(1-131aa)-		GCGCGGATCCGAAATCCATGTCGCAGCC
BamHl VDAC1(132-283aa)-Xhol	GCGCCTCGAGCTGACATTGCTGGGCCTTCC	
VDAC1 K34R	CTTGATTTGAAAACAAGATCTGAGAATG	CTTGTTTTCAAATCAAGCTTTATTAAG
VDAC1 K109R	TCACCTAACACTGGGAGAAAAAATGCTA	CTCCCAGTGTTAGGTGAGAAGGATGAA
VDAC1 K110R FP	CCTAACACTGGGAAAAGAAATGCTAAAA	CTTTTCCCAGTGTTAGGTGAGAAGGAT
VDAC1 K161R	AATTTTGAGACTGCAAGATCCCGAGTGA	CTTGCAGTCTCAAAATTCATCTGGTAG
VDAC1 K197R	GGCTCCATTTACCAGAGAGTGAACAAGA	CTCTGGTAAATGGAGCCGCCAAACTCT
VDAC1 K200R FP	TACCAGAAAGTGAACAGGAAGTTGGAGA	CTGTTCACTTTCTGGTAAATGGAGCCG
VDAC1 K201R FP	CAGAAAGTGAACAAGAGGTTGGAGACCG	CTCTTGTTCACTTTCTGGTAAATGGAG
VDAC1 K252R	CCTGCTTCTCGGCTAAGGTGAACAACTC	CTTAGCCGAGAAGCAGGCGTCAGGGTC
VDAC1 K266R	GCTCTTCTGGATGGCAGGAACGTCAATG	CTGCCATCCAGAAGAGCTGACAGTGTC
mβ-actin	GATATCGCTGCGCTGGTCG	CATTCCCACCATCACACCCT
